# Type I IFN stimulates IFI16-mediated aromatase expression in adipocytes that promotes E_2_-dependent growth of ER-positive breast cancer

**DOI:** 10.1007/s00018-022-04333-y

**Published:** 2022-05-20

**Authors:** Na-Lee Ka, Ga Young Lim, Seung‑Su Kim, Sewon Hwang, Juhyeong Han, Yun-Hee Lee, Mi-Ock Lee

**Affiliations:** 1 College of Pharmacy, Seoul National University, Seoul, 08826 South Korea; 2 Research Institute of Pharmaceutical Sciences, Seoul National University, Seoul, 08826 South Korea; 3 Bio-MAX Institute, Seoul National University, Seoul, 08826 South Korea

**Keywords:** Aromatase, Breast cancer, Cancer-associated adipocytes, Estrogen signaling, IFI16, Type 1 interferon

## Abstract

Although type I interferons (IFNs) play multifaceted roles during tumorigenesis and cancer treatment, the interplay between type I IFNs and estrogen signaling in breast cancer (BC) microenvironment is not well understood. Here, we report a novel function of type I IFNs in inducing aromatase expression in adipose tissues surrounding BC, which potentiates the E_2_-dependent growth of estrogen receptor (ER)-positive BC. First, we found that expression levels of type I IFNs correlate negatively with clinical outcome but positively with tumor grade in patients with ER-positive BC. Levels of type I IFNs were elevated in cocultured media of immune cells and BC cells, which increased aromatase expression and E_2_ production in Simpson–Golabi–Behmel syndrome preadipocytes. The type I IFN-induced aromatase expression was dependent on IFN-γ-inducible protein 16 (IFI16), which is encoded by an interferon-stimulated gene. At the molecular level, type I IFNs led to recruitment of HIF1α–IFI16–PRMT2 complex to the hypoxia-response element located in the aromatase PI.3/PII promoter. Next, we generated an adipocyte-specific Ifi204, which is a mouse ortholog of human IFI16, knockout mouse (Ifi204-AKO). IFNβ induced E_2_ production in the preadipocytes isolated from the control mice, but such E_2_ production was far lower in the Ifi204-AKO preadipocytes. Importantly, the growth of orthotopically inoculated E0771 ER-positive mammary tumors was reduced significantly in the Ifi204-AKO mice. Taken together, our findings provide novel insights into the crosstalk between type I IFNs and estrogen signaling in the progression of ER-positive BC.

**Supplementary Information:**

The online version contains supplementary material available at 10.1007/s00018-022-04333-y.

## Introduction

Among women, breast cancer (BC) is the most frequently diagnosed cancer and the leading cause of cancer-related death worldwide [1]. Approximately, 75% of BCs show expression of estrogen receptor (ER), which drives E_2_-dependent tumor growth and disease progression. The majority of BCs occur in postmenopausal women over the age of 50 [2]. After menopause, when the ovaries no longer produce substantial amounts of estrogens, the circulating levels of estrogens become low. However, the concentrations of estrogens in breast tumors are at least 20-fold greater than those in the circulation, probably due to locally synthesized estrogens in extragonadal sites, such as adipose tissues surrounding the breast tumor [3]. Importantly, obesity (body mass index ≥ 30 kg/m^2^) is associated with an increased risk of ER-positive BC in postmenopausal women [4]. Elevated serum estrogen levels and increased local production of estrogen with increasing body mass index have been considered as primary mediators of BC development in postmenopausal women [5]. Moreover, obesity impairs efficacy of several components of BC treatment and increases the risk of recurrence and cancer-associated death [6]. Therefore, the development of effective strategies to reduce local estrogen production in patients with BC and obesity is highly important, especially in postmenopausal women.

Estrogens are synthesized from androgens in a reaction catalyzed by the enzyme aromatase, which is encoded by the cytochrome P450 family 19 subfamily A member 1 (CYP19A1) gene. Transcription of the aromatase gene is controlled by several distinct and tissue-specific promoters, each of which is regulated by different hormones, cytokines, and second messenger signaling pathways [7, 8]. Transcripts of aromatase in different tissues contain unique untranslated first exons driven by the alternative promoters, which are spliced onto the coding exon II at the common splice site immediately upstream of the ATG translation start site [7]. Under disease-free conditions, aromatase expression is predominantly driven by the relatively weak promoter I.4 in breast adipose tissues. However, the main promoter is switched to more potent promoters I.3 and PII in breast adipose tissues in the presence of tumors, which leads to a marked increase in aromatase expression [7, 8]. Proinflammatory mediators, such as prostaglandin E_2_, secreted from obesity-linked inflammatory adipose tissues, increased expression of aromatase through induction of the PII promoter, which may lead to the main promoter switch [9]. A recent study has shown that obesity induces type I interferon (IFN) signaling in adipocytes, which promotes adipocyte inflammation and pathogenesis of obesity-associated sequelae [10]. Based on the potential interconnection between adipose inflammation and aromatase activation in BC microenvironment, identification of obesity-associated inflammatory signaling pathways that affect local estrogen production may provide insights into inhibition of growth of ER-positive BCs.

The type I IFNs, predominantly IFNα and IFNβ, constitute a family of cytokines with pleiotropic effects that modulate the immune response against viral infections, autoimmune diseases, and cancers. Owing to the effectiveness of type I IFNs in antitumor immunity, many attempts have been made to explore their potential use in treatment of various cancers [11]. In BC, antitumor effects of type I IFNs have been evaluated particularly in triple negative BC (TNBC) because of its high immunogenicity [12]. Recently, we reported that type I IFNs induce antitumor immunity in TNBC through induction of STING signaling autoactivation particularly after chemotherapy [13]. However, numerous studies have reported contradictory findings that type I IFNs promote growth and progression of BCs. For example, type I IFNs were upregulated in inflammatory BCs and contributed to the protumorigenic milieu [14]. Moreover, IFNs upregulated survival factors, such as G1P3, which promoted tumor cell survival and contributed to poor outcomes in ER-positive BCs [15]. IFNs also induced a subset of IFN-stimulated genes (ISGs), which were identified as an IFN-related DNA damage-resistant signature conferring resistance to therapy in patients with BC [16]. These inconsistent effects of type I IFNs on different BC types may depend on the tumor microenvironment, such as the differential composition of stroma cells including cancer-associated adipocytes, and IFN-responding intracellular contexts, such as the ISGs. We previously reported that one of the ISGs, IFN-γ-inducible protein 16 (IFI16), was differentially expressed between ER-positive and ER-negative BCs and that it positively or negatively regulated ER expression depending on the cellular context of the BC [17]. Taken together, these studies highlight the importance of cellular and environmental contexts of the BC microenvironment in type I IFN signaling and its therapeutic consequences. Thus, we aimed to illustrate a potential role of type I IFNs in BC microenvironment that affects the expression of aromatase in cancer-associated adipocytes and the subsequent E_2_-dependent growth of ER-positive BC.

## Results

### Expression levels of type I IFNs correlate negatively with clinical outcome but positively with tumor grade in patients with ER-positive BC

**Fig. 1:**
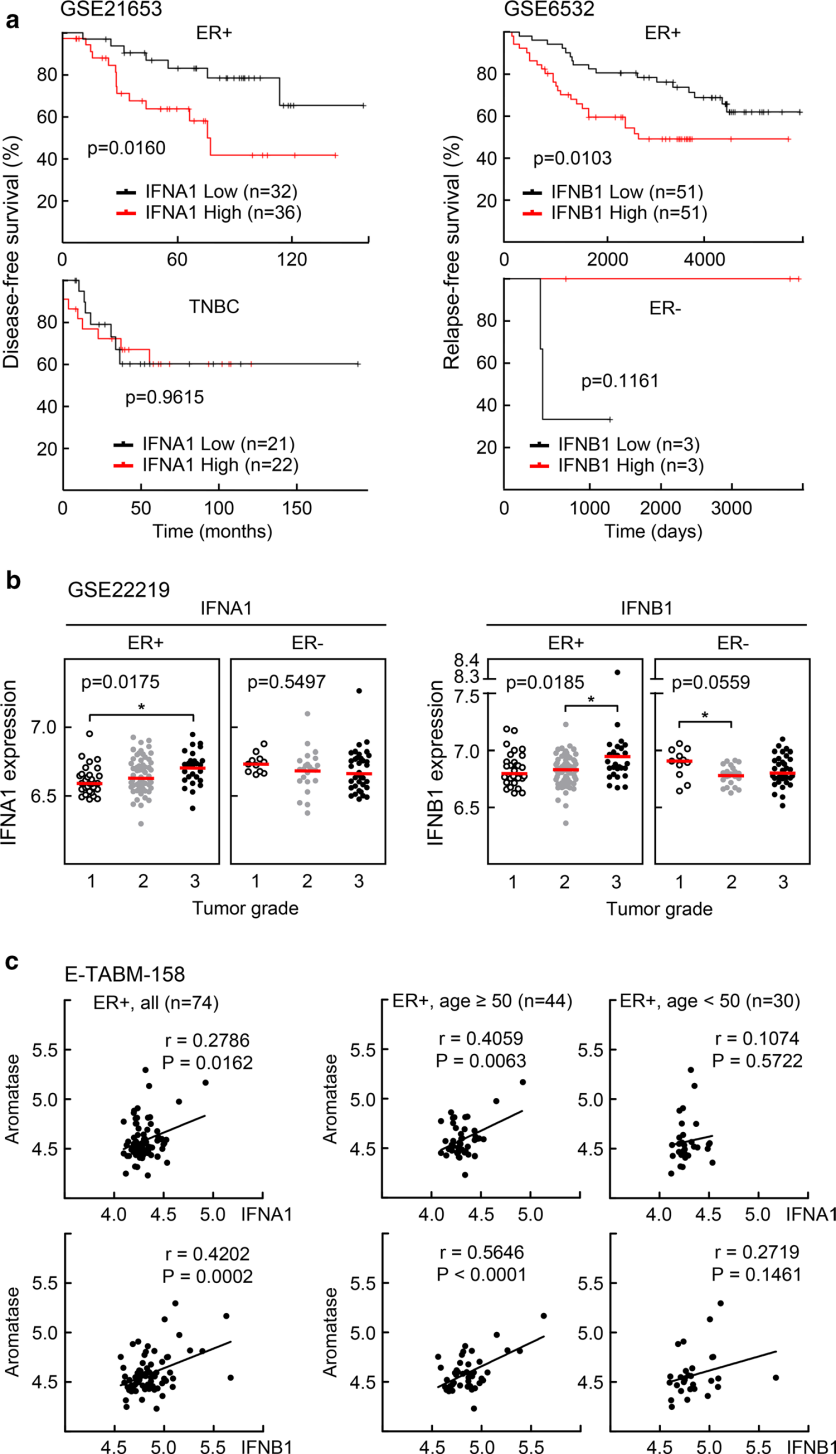
Clinical consequences of BC patients with different IFN expression levels. **a** The GSE21653 and the GSE6532 datasets were obtained and analyzed in the CTGS website (http://ctgs.biohackers.net/). Kaplan–Meier analysis was carried out in the group of patients stratified by ER status. Patients were categorized into a low (lower quartile) and a high (upper quartile) IFN expression groups. To analyze statistical differences, Log-rank (Mantel–Cox) tests were performed. **b** Gene expression analysis was performed using GSE22219 dataset obtained from the NCBI GEO website. Expression levels of IFNA1 and IFNB1 were analyzed in different grades of BC specimens stratified by ER status. Bars in red indicate the median expression level in each group. Statistical analysis was performed using one-way ANOVA followed by Tukey’s HSD post hoc test. **c** Gene expression analysis was conducted using E-TABM-158 dataset obtained from ArrayExpress website. A case that lacked age information record was excluded from data analyses. Correlation between the expression of IFN and aromatase was analyzed in ER-positive BC patients by age group. Pearson’s correlation coefficient (*r*) was calculated

We were interested in understanding the cellular and environmental contexts of the BC microenvironment that led to inconsistent effects of type I IFNs on different types of BC. We first analyzed the expression levels of type I IFNs and clinical outcome in BC cohorts using publicly available datasets, GSE21653 and GSE6532 [18, 19]. We found that the high expression of both IFNA1 and IFNB1 was associated with a worse clinical outcome of patients with ER-positive BC but not of patients with ER-negative BC (Fig. 1a). In addition, expression level of IFNA1 and IFNB1 increased with tumor grade in case of ER-positive BC, whereas it decreased in case of ER-negative BC (Fig. 1b) [20]. Interestingly, mRNA expression levels of IFNA1 and IFNB1 were closely correlated with aromatase expression in ER-positive BC, particularly in patients with over 50 years of age who might have been through the menopause (Fig. 1c) [21]. These findings suggest that the negative effect of type I IFNs on the progression of ER-positive BC may be associated with the local production of estrogen in the tumor microenvironment.

### Type I IFNs increase E_2_ production through transcriptional induction of aromatase in Simpson–Golabi–Behmel syndrome preadipocytes

**Fig. 2:**
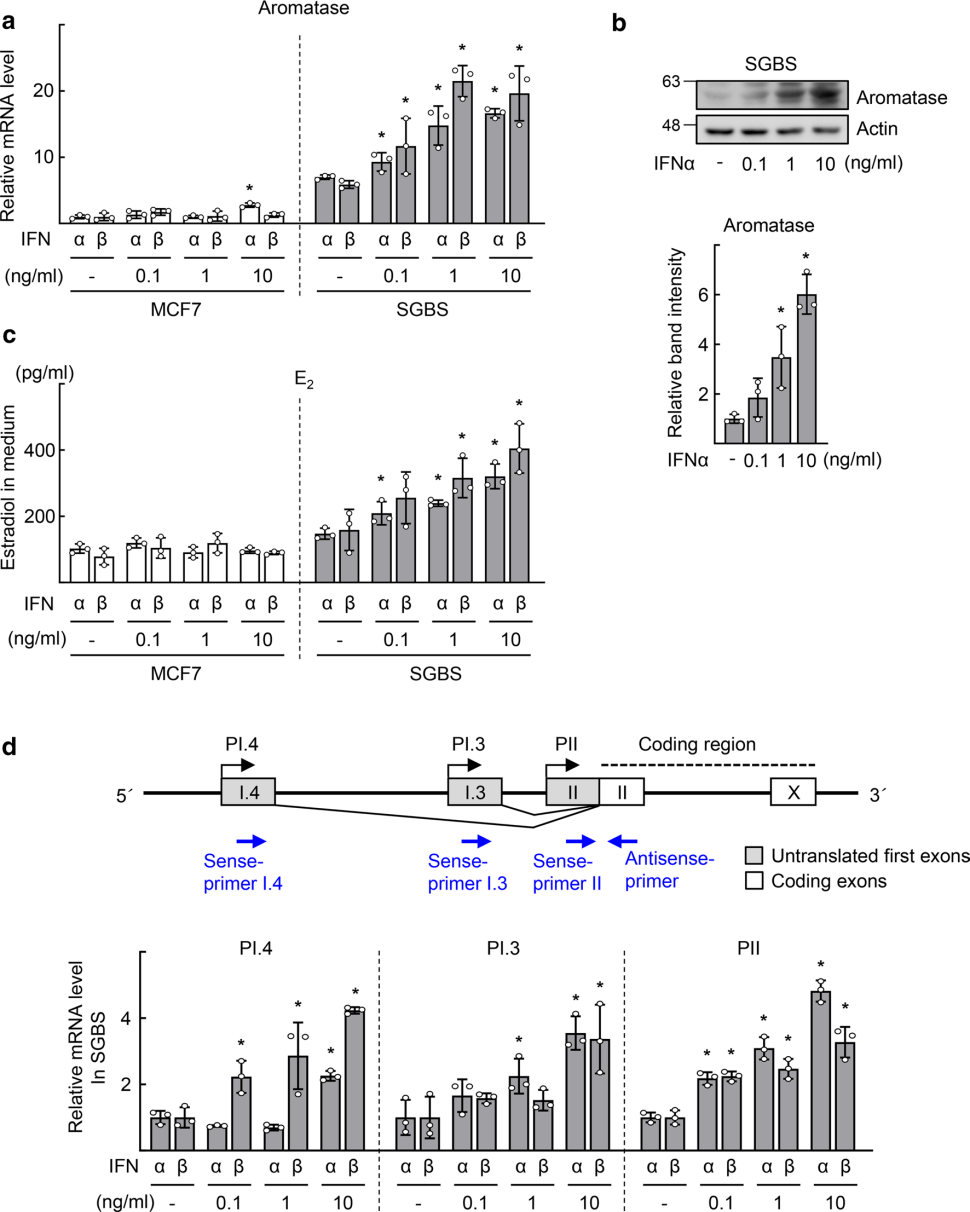
Type I IFN leads to transcriptional activation of aromatase and E_2_ production in the SGBS cells. **a** MCF7 (4 × 10^5^ cells) or SGBS cells (2 × 10^5^ cells) were treated with vehicle (−), IFNα, or IFNβ for 48 h. The doubling times of MCF7 and SGBS without IFN stimulation were about 25 h and 23 h, respectively. The mRNA level of aromatase was measured by qPCR. **b** SGBS cells (2 × 10^5^ cells) were treated with vehicle (−) or IFNα for 48 h, as indicated. Expression level of aromatase was analyzed by western blotting (top). Band intensity of aromatase was quantified using ImageJ and normalized to that of Actin band (bottom). **c** MCF7 (4 × 10^5^ cells) or SGBS (2 × 10^5^ cells) cells were treated with vehicle (-), IFNα, or IFNβ for 48 h, as indicated. The concentration of E_2_ in the culture supernatants was measured by ELISA. **d** Structure of aromatase gene promoters and primers used for measurement of promoter activity (top). SGBS cells were treated with vehicle (−), IFNα, or IFNβ for 48 h. The levels of the PI.4, PI.3, or PII-specific aromatase transcripts were measured by qPCR (bottom). Data are presented as the mean ± SD (*n* = 3). *P < 0.05 vs vehicle

In postmenopausal women, aromatase in adipose tissues surrounding the breast tissue plays a critical role in supplying estrogen to BC [3, 22]. Therefore, we asked whether IFNα and IFNβ affect the expression of aromatase in adipose tissues. To answer the question, we employed the Simpson–Golabi–Behmel syndrome (SGBS) preadipocyte cell strain, a representative model of human preadipocytes [23]. Particularly, aromatase expression and activity in SGBS cells were reported to be similar to those in isolated human preadipocytes [24]. mRNA level of aromatase was enhanced by both IFNα and IFNβ in SGBS cells, however, it was not or marginally increased in MCF7, T47D, and BT474, which are ER-positive BC cell lines (Figs. 2a and S1a). Consistently, the level of aromatase protein as well as production of estrogen increased by IFNα or IFNβ treatment in SGBS preadipocytes but not in the ER-positive BC cell lines (Figs. 2b and c and S1b). Among the multiple tissue-specific promoters of aromatase, promoters I.4 (PI.4), I.3 (PI.3), and II (PII) were responsible for the expression of aromatase in adipose tissues adjacent to BC. The PI.3 and PII share common cis-regulatory elements since the TATA boxes of these promoters are separated by only 215 bp; thus called often as PI.3/PII [7]. Quantitative real-time polymerase chain reaction analysis using promoter-specific primers demonstrated that both IFNα and IFNβ increased expression of aromatase transcripts via the activation of PI.4, PI.3, and PII in SGBS cells (Fig. 2d). Type I IFN-induced aromatase expression and E_2_ production were also observed in differentiated SGBS adipocytes (Fig. S1c).

In the tumor microenvironment, type I IFNs are produced by tumor-infiltrating immune cells as well as malignant cells [25, 26]. Macrophages are prominent in the stromal compartment and represent a major cell population among the infiltrating immune cells, constituting over 50% of the tumor mass in BC [27]. Therefore, we examined whether type I IFNs released by BC cells and macrophages are able to induce aromatase expression and the subsequent production of estradiol in cultured SGBS preadipocytes. Differentiated THP1 macrophages or MCF7 cells were cultured individually (monoculture) or cultured together (coculture) in a transwell culture system (Fig. S2a). Interestingly, secretion of IFNα and IFNβ increased dramatically when THP1 and MCF7 cells were cocultured (Fig. S2a). The basal mRNA levels of IFNα and IFNβ were higher in THP1 cells than in MCF7 cells when these cells were monocultured, but they increased by two- to threefold in both cell types when these cells were cocultured (Fig. S2b). Next, we measured estradiol production in SGBS preadipocytes cultured using conditioned media (CM) obtained from monocultured or cocultured THP1 and MCF7 cells. Consistent with the levels of type I IFNs, the CM obtained from the cocultured plates increased E_2_ production in SGBS cells to the highest level (Fig. S2c). The aromatase expression level which was induced by the CM from the cocultured plates was attenuated by addition of neutralizing antibodies against IFNα and IFNβ, indicating type I IFN-induced aromatase expression (Fig. S2d).

### IFI16 mediates type I IFN-induced aromatase transcription by binding to PI.3/PII promoter

**Fig. 3:**
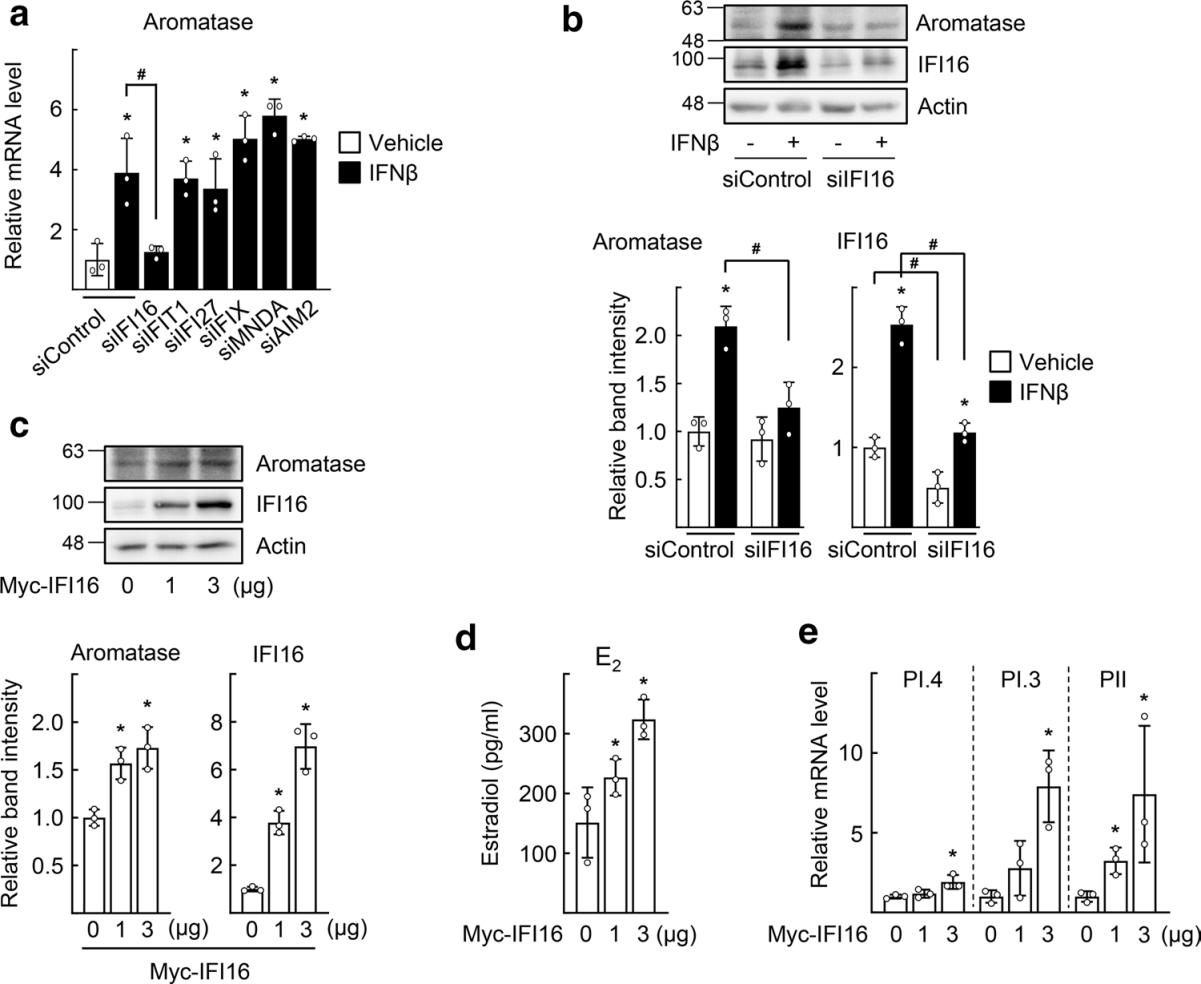
IFI16 mediates the type I IFN-induced upregulation of aromatase in SGBS cells. **a** SGBS cells were transfected with the indicated siRNAs. Twenty-four hours after transfection, the cells were treated with 10 ng/ml IFNβ for 48 h. Expression level of aromatase was measured by qPCR. *P < 0.05 vs siControl with vehicle, ^#^*P* < 0.05 as indicated (*n* = 3). The mRNA levels of ISGs are shown as control in Fig. S3. **b** SGBS cells were transfected with control siRNA or IFI16 siRNA. Twenty-four hours after transfection, the cells were treated with 10 ng/ml IFNβ for 48 h. Expression levels of protein were analyzed by western blotting (top). Band intensities of aromatase and IFI16 in blots were quantified using ImageJ and normalized to that of Actin band (bottom). **c**–**e** SGBS cells were transfected with empty vector (0) or the indicated amount of Myc-IFI16 for 48 h. Expression level of aromatase was analyzed by western blotting (**c**, top). Band intensities of aromatase and IFI16 in blots were quantified using ImageJ and normalized to that of Actin band (**c**, bottom). The concentration of estradiol in the culture medium was measured by ELISA (**d**). Expression levels of the PI.4, PI.3, or PII-specific aromatase transcripts were measured by qPCR (**e**). *P < 0.05 vs empty vector (0) (*n* = 3)

Next, we sought to identify the potential molecular mechanism by which type I IFNs induce aromatase transcription in SGBS cells. First, to identify ISGs that mediate the effects of type I IFNs, six known ISGs, including IFI16 and AIM2, were knocked down. Only depletion of IFI16 reduced the IFNβ-induced expression of aromatase transcripts (Figs. 3a and S3). Depletion of IFI16 also attenuated the IFNβ-induced protein level of aromatase (Fig. 3b). Consistently, overexpression of IFI16 increased the expression of aromatase and estradiol production in SGBS preadipocytes (Fig. 3c and d). Furthermore, the levels of PI.3 and PII promoter-specific aromatase transcripts greatly increased by IFI16 overexpression, suggesting that IFI16 may mediate type I IFN-induced aromatase expression via its action on PI.3 and PII aromatase promoters (Fig. 3e).

**Fig. 4:**
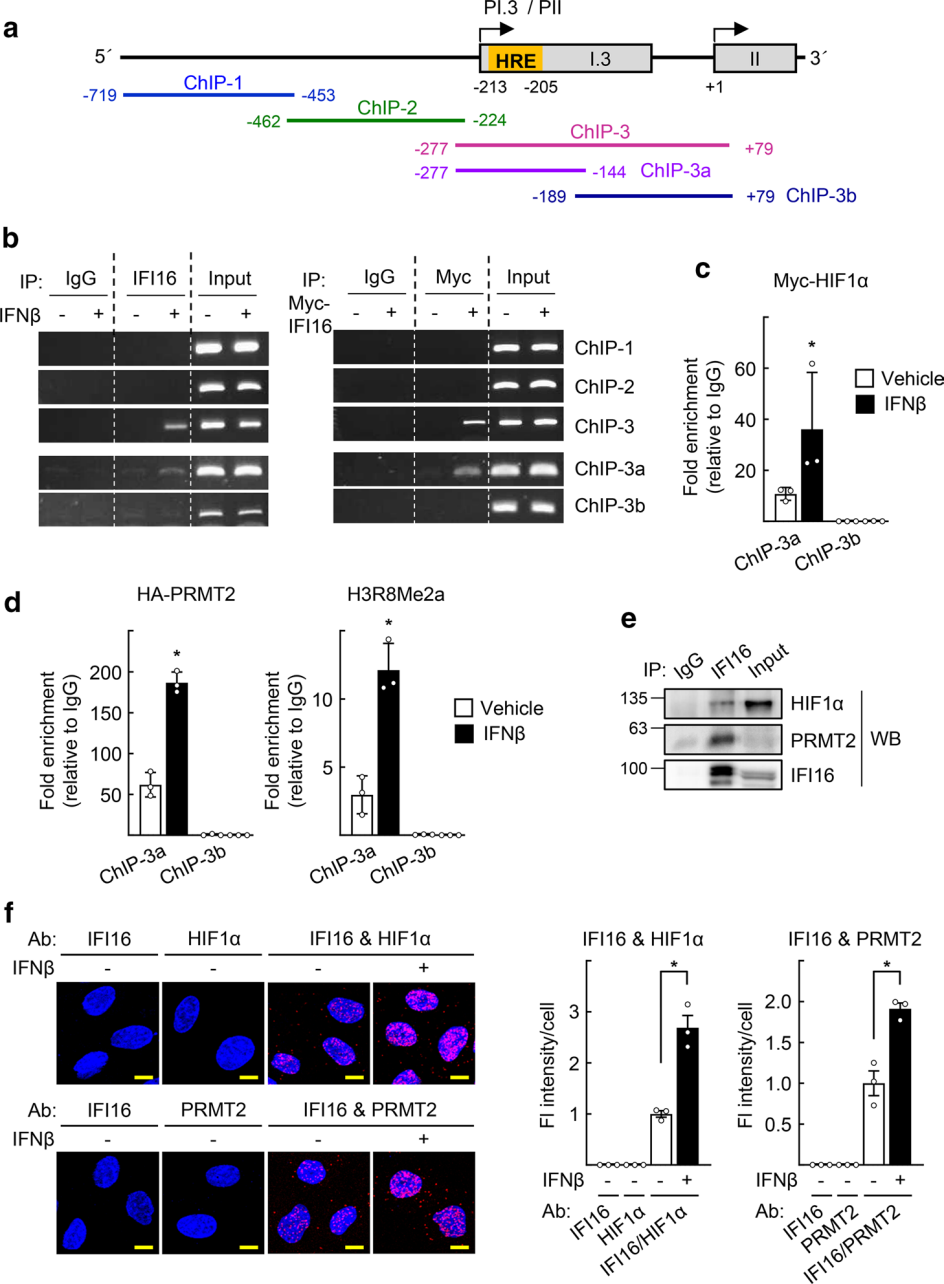

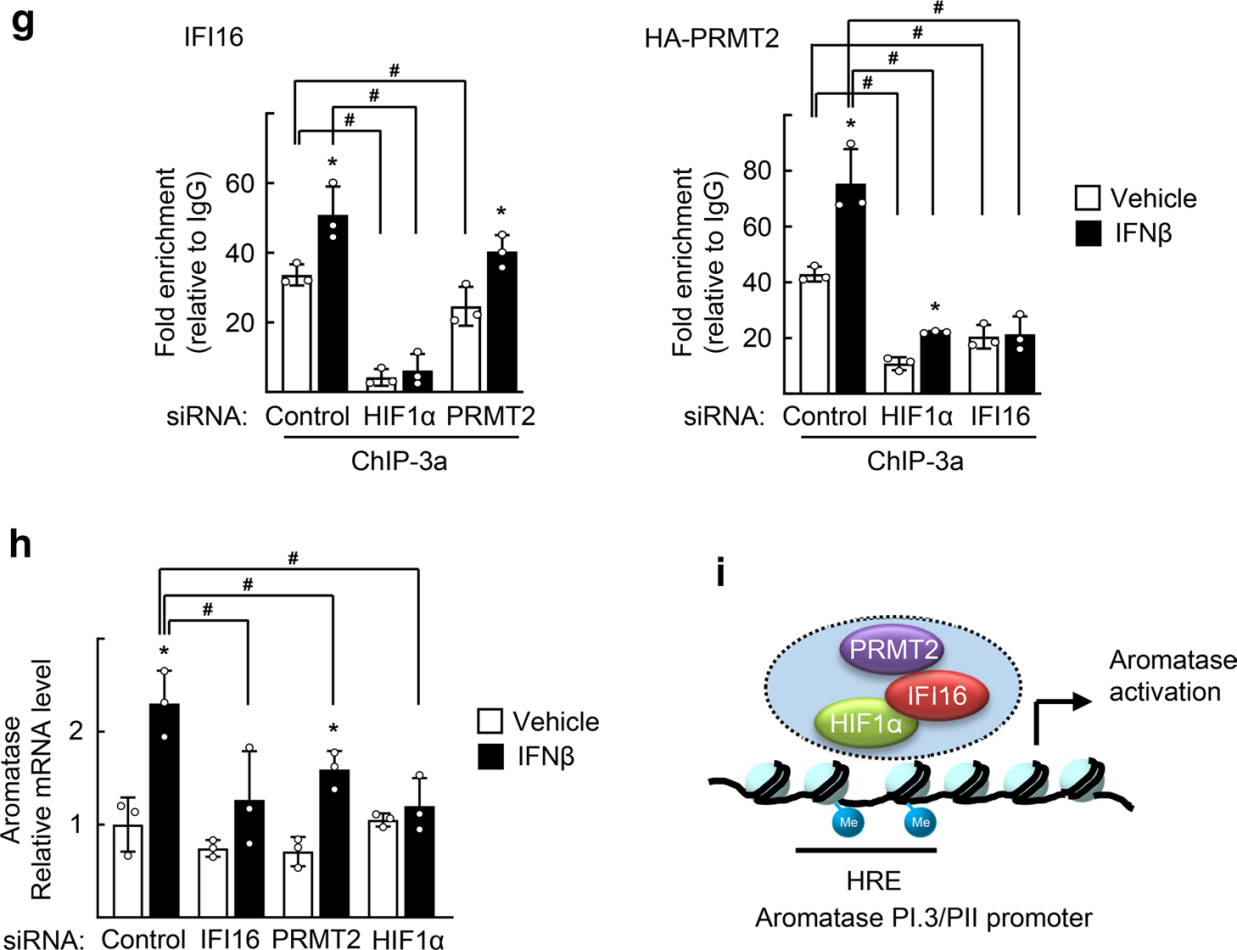
IFI16 activates aromatase PI.3/PII promoter through DNA binding together with HIF1α and PRMT2. **a** Schematic representation of aromatase PI.3 and PII promoters and primers used for ChIP experiments. **b** SGBS cells were treated with 10 ng/ml IFNβ (left) or transfected with Myc-IFI16 (right) for 48 h. Cells were fixed and subjected to ChIP assay using anti-IFI16 or anti-Myc antibodies. Immunoprecipitated DNA fragments were amplified by PCR analysis using the indicated primers. **c** SGBS cells were transfected with Myc-HIF1α and treated with 10 ng/ml IFNβ for 48 h. Cells were fixed and subjected to ChIP assay using anti-Myc antibody, followed by qPCR analysis with the indicated primers. *P < 0.05 vs vehicle (*n* = 3). **d** SGBS cells were transfected with HA-PRMT2 and treated with 10 ng/ml IFNβ for 48 h. Cells were fixed and subjected to ChIP assay using anti-HA or anti-H3R8Me2a antibodies, followed by qPCR analysis with the indicated primers. *P < 0.05 vs vehicle (*n* = 3). **e** SGBS cells were transfected with Myc-HIF1α. Whole cell lysates were immunoprecipitated (IP) using IgG or anti-IFI16 antibodies and probed with the indicated antibodies by western blotting (WB). **f** SGBS cells were treated with 10 ng/ml IFNβ for 48 h. Interactions of IFI16-HIF1α and IFI16-PRMT2 were visualized with red dots by in situ proximity ligation assay. As a negative control, a single staining with the anti-IFI16, anti-HIF1α, or anti-PRMT2 antibodies was performed. DAPI was used to stain the nuclei (left). The fluorescence (Fl) intensity per cell was quantified from at least 100 cells using ImageJ software (right). Data are presented as the mean ± SEM. *P < 0.05 (*n* = 3). Bar represents 10 μm. **g** SGBS cells were transfected with the indicated siRNAs (left) or siRNA together with HA-PRMT2 (right), and treated with 10 ng/ml IFNβ for 48 h. Cells were fixed and subjected to ChIP assay using anti-IFI16 or anti-HA antibodies, followed by qPCR analysis with the indicated primers. *P < 0.05 vs vehicle, ^#^*P* < 0.05 vs siControl (*n* = 3). **h** SGBS cells were transfected with indicated siRNAs and treated with 10 ng/ml IFNβ for 48 h. Expression level of aromatase was measured by qPCR. *P < 0.05 vs vehicle, ^#^*P* < 0.05 vs siControl treated with IFNβ (*n* = 3). The mRNA levels of IFI16, PRMT2, and HIF1α are shown as control in Fig. S4. **i** Schematic illustration for binding of HIF1α-IFI16-PRMT2 complex to the HRE on aromatase PI.3/PII promoter that activates aromatase transcription in preadipocytes

To further characterize the role of IFI16 with regard to the aromatase promoters, we examined whether IFI16 binds to PI.3 and PII using chromatin immunoprecipitation analysis (Fig. 4a). We found that IFI16 was recruited to a region (− 277 to − 144) that is located in PI.3 (Fig. 4b). Interestingly, it was previously reported that this region encoded a hypoxia-response element (HRE, − 213 to − 205) [28]. We also observed that HIF1α bound to the HRE and that this binding was enhanced after IFNβ treatment (Fig. 4c). Next, we tested whether protein arginine N-methyltransferase 2 (PRMT2) bound to the HRE because we previously observed that PRMT2 and IFI16 were recruited to the same DNA region [29]. Surprisingly, PRMT2 bound the HRE site, and this binding was enhanced by IFNβ treatment (Fig. 4d). We further observed that the level of H3R8me2a, an activation marker produced by PRMT2-mediated catalysis, increased after IFNβ treatment (Fig. 4d). Indeed, IFI16 formed a complex with PRMT2 and HIF1α, as revealed by coimmunoprecipitation experiments (Fig. 4e). The association of IFI16 with HIF1α and PRMT2 was increased after IFNβ treatment, when examined using in situ proximity ligation assay (PLA) (Fig. 4f). The binding of IFI16 to the HRE was attenuated by a knockdown of HIF1α but not that of PRMT2, whereas the binding of PRMT2 was attenuated by a knockdown of IFI16 (Fig. 4g). This finding may suggest a sequential recruitment of HIF1α, IFI16, and PRMT2 to the PI.3/PII promoter. Furthermore, depletion of IFI16, PRMT2, or HIF1α by small interfering RNA attenuated the IFNβ-induced aromatase expression, supporting the importance of the HIF1α–IFI16–PRMT2 complex in this process (Figs. 4h and S4). Taken together, these findings demonstrate that type I IFNs lead to the binding of the HIF1α–IFI16–PRMT2 complex to the HRE site on the aromatase PI.3/PII promoter for induction of aromatase expression (Fig. 4i).

### Type I IFN/IFI16 pathway in adipocytes supports the growth of ER-positive BC

**Fig. 5:**
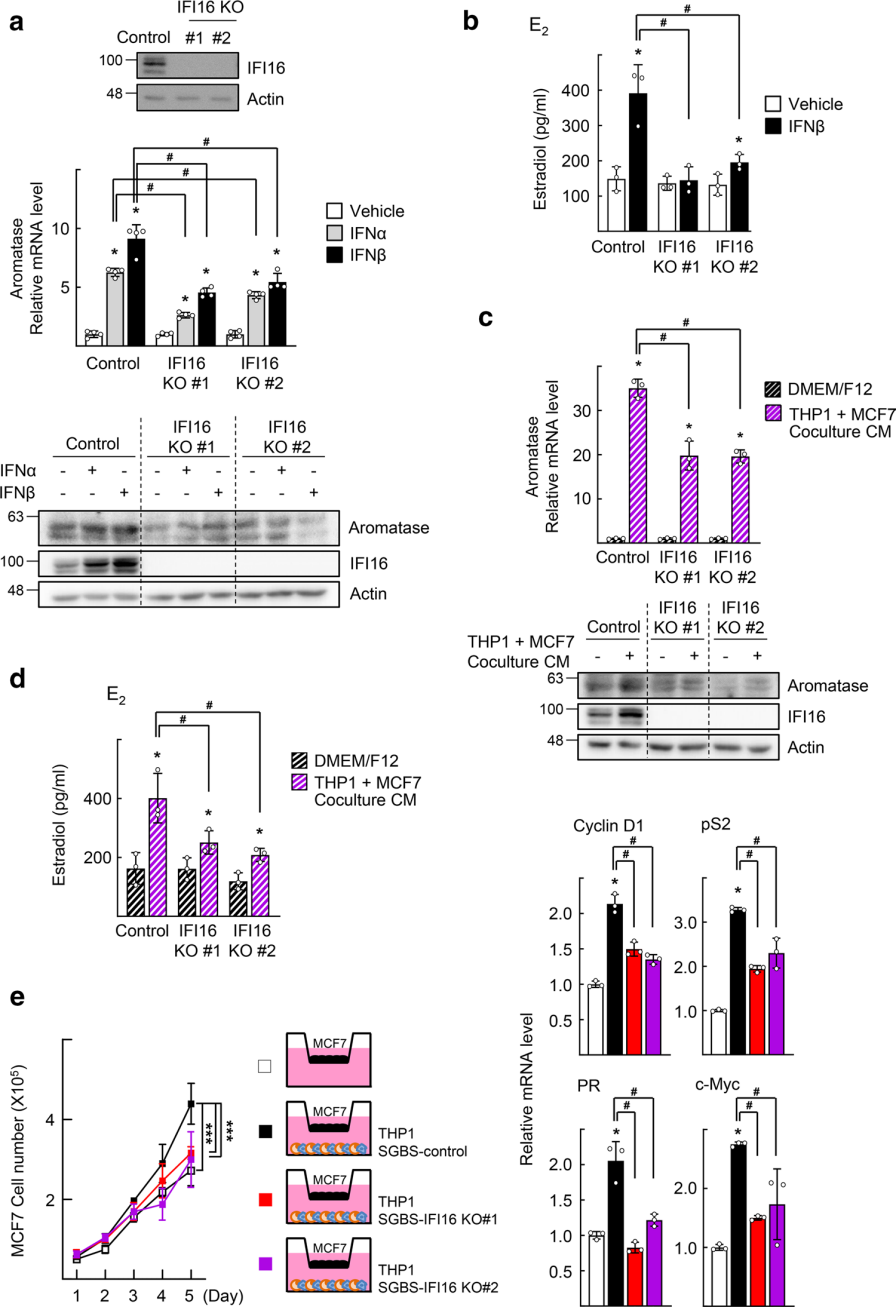
E_2_-dependent growth of MCF7 is decreased in conditioned media obtained from the IFI16 KO SGBS preadipocytes. **a** Expression level of IFI16 in SGBS control or IFI16 KO cells was analyzed by western blotting (top). SGBS control or IFI16 KO cells were treated with 10 ng/ml IFNs for 48 h. Expression levels of mRNA and protein of aromatase were analyzed by qPCR (middle) and western blotting (bottom), respectively. *P < 0.05 vs vehicle, ^#^*P* < 0.05 vs control treated with IFN (*n* = 4). **b** SGBS control or IFI16 KO cells were treated with 10 ng/ml IFNβ for 48 h. The concentration of E_2_ in the culture supernatants was measured by ELISA. *P < 0.05 vs vehicle, ^#^*P* < 0.05 vs control treated with IFNβ (*n* = 3). **c, d** THP1 and MCF7 cells were cocultured as described in Fig. S2a. SGBS control or IFI16 KO cells were incubated with conditioned medium (CM) obtained from the THP1 and MCF7 coculture for 48 h. Expression levels of mRNA and protein of aromatase were analyzed by qPCR (top) and western blotting (bottom), respectively (**c**). The concentrations of E_2_ in the culture medium were measured by ELISA (**d**). *P < 0.05 vs DMEM/F12, ^#^*P* < 0.05 vs control with coculture CM (*n* = 3). **e** THP1 cells pre-treated with 100 ng/ml PMA for 48 h, and SGBS cells were seeded in the bottom chamber of the transwell, as indicated in the scheme. MCF7 cells were seeded in the top chamber of the transwell insert. The number of viable MCF7 cells was counted using a hemocytometer. Statistical analysis was performed using two-way ANOVA followed by the Bonferroni posttest. ***P < 0.001 vs MCF7 cocultured with SGBS control with THP1 cells (filled square) (*n* = 4) (left). Total RNAs were obtained from the MCF7 cells at the day 5 and subjected to qPCR analysis. *P < 0.05 vs MCF7 monoculture, ^#^*P* < 0.05 vs MCF7 cocultured with SGBS control with THP1 cells (filled square) (*n* = 3) (right)

We examined whether the type I IFN/IFI16-induced E_2_ production in adipocytes affected the growth of ER-positive BC cells. We established two SGBS sublines lacking IFI16 (IFI16 knockout [KO] #1 and #2) using the CRISPR/Cas9 system. Type I IFN-induced aromatase expression and E_2_ production was attenuated in SGBS-IFI16 KO sublines (Fig. 5a and b). In addition, the CM obtained from THP1/MCF7 cocultured plates significantly decreased aromatase expression and E_2_ production in SGBS-IFI16 KO cells to compare with those in SGBS-control cells (Fig. 5c and d). To determine whether the knocking out of IFI16 in SGBS cells affected the growth of MCF7 cells when cocultured, the growth of MCF7 cells was measured after coculture with THP1 and SGBS cells. The growth of MCF7 cells was significantly slower when cocultured with SGBS-IFI16 KO cells than with SGBS-control cells (Fig. 5e). Expression of the ER target genes, such as cyclin D1, pS2, progesterone receptor, and c-Myc, was significantly attenuated when cocultured with SGBS-IFI16 KO cells (Fig. 5e). These findings indicate that IFI16 in SGBS cells supports the growth of ER-positive BC cells, probably by providing E_2_ in the culture media.

**Fig. 6:**
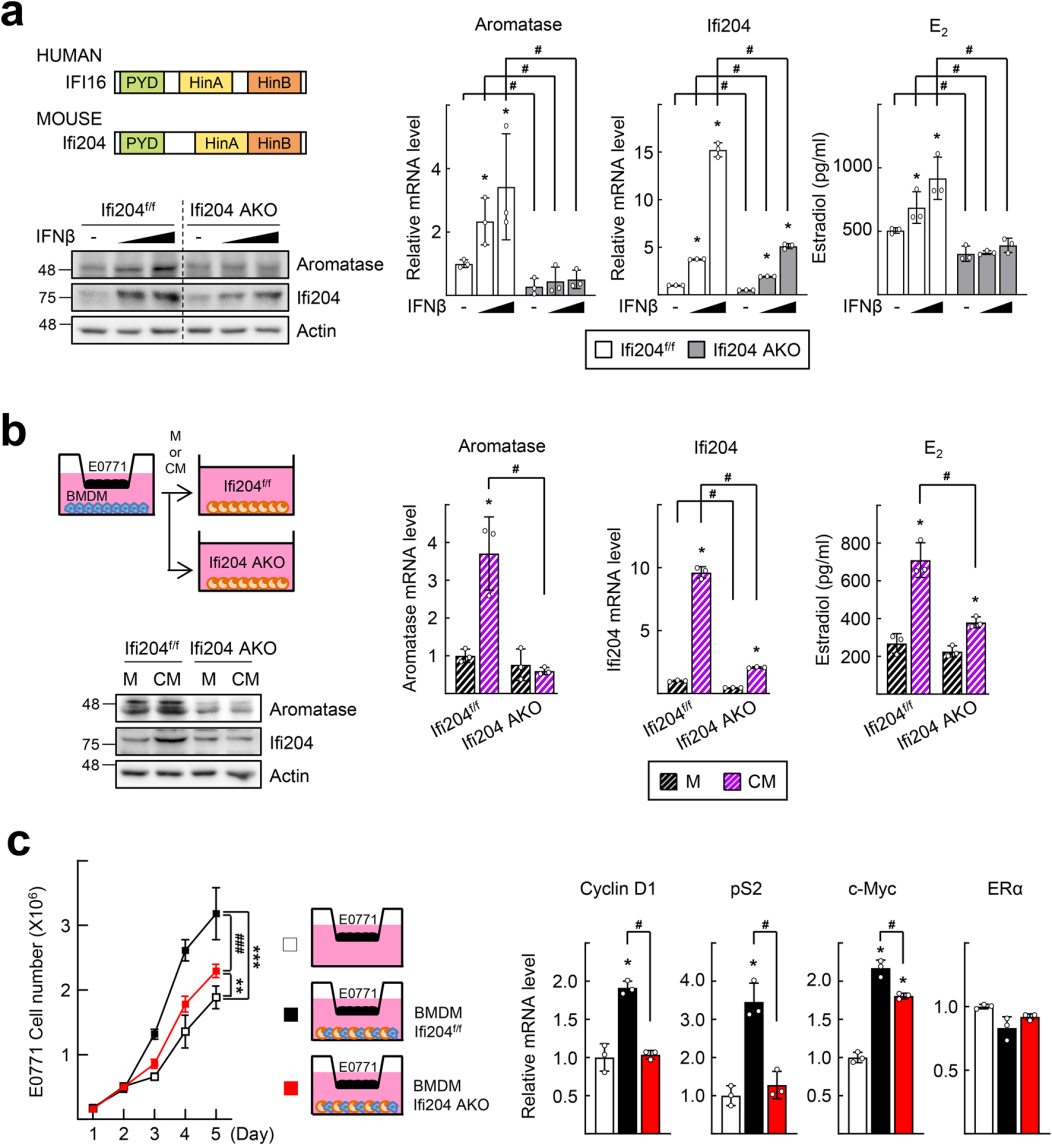
Adipocyte-specific knockdown of Ifi204 attenuates the type I IFN-induced upregulation of aromatase and E_2_ production. **a** Schematic presentation of the domain structure of human IFI16 and mouse Ifi204 proteins. Both proteins comprise a pyrin domain (PYD) involved in protein–protein interaction, and two hematopoietic interferon-inducible nuclear (Hin) domains, HinA and HinB, which are involved in DNA binding (left top). Primary preadipocytes isolated from the inguinal mammary gland of Ifi204-AKO and Ifi204^f/f^ mice were treated with vehicle (−), or 1 or 10 ng/ml IFNβ for 48 h. Expression levels of aromatase and Ifi204 were measured by western blotting (left bottom) and qPCR (right). The concentration of E_2_ in the culture supernatants was measured by ELISA (right). *P < 0.05 vs vehicle, ^#^*P* < 0.05 vs Ifi204^f/f^ (*n* = 3). **b** Schematic representation of the E0771 and BMDM coculture experiments (left top). BMDMs were seeded in the bottom chamber of a transwell and E0771 cells were seeded in the top chamber and allowed to incubate for additional 48 h. Primary preadipocytes were incubated with DMEM (M) or conditioned medium (CM) obtained from the E0771 and BMDM coculture for 48 h. Expression levels of aromatase and Ifi204 were measured by western blotting (left bottom) and qPCR (right). The concentration of E_2_ in the culture supernatants was measured by ELISA (right). *P < 0.05 vs DMEM, ^#^*P* < 0.05 vs Ifi204^f/f^ (*n* = 3). **c** Mouse BMDMs and primary preadipocytes were seeded in the bottom chamber of the transwell, as indicated in the scheme. E0771 cells were seeded in the top chamber of the transwell insert. The number of viable E0771 cells was counted using a hemocytometer. Statistical analysis was performed using two-way ANOVA followed by the Bonferroni posttest. ***P < 0.001 vs E0771 monoculture, ^###^*P* < 0.001 vs E0771 cocultured with Ifi204^f/f^ preadipocytes with BMDMs (filled square) (*n* = 4) (left). Total RNAs were obtained from the E0771 cells at the day 5 and subjected to qPCR analysis. *P < 0.05 vs E0771 monoculture, ^#^*P* < 0.05 vs E0771 cocultured with Ifi204^f/f^ preadipocytes with BMDMs (filled square) (*n* = 3) (right)

Next, we expanded this study to a member of the mouse Ifi200 family of IFN-inducible genes, Ifi204, which is a mouse ortholog of human IFI16 [30] (Fig. 6a). We generated an adipocyte-specific Ifi204 KO mouse, Ifi204-AKO, by crossbreeding of the Ifi204^f/f^ and the fatty acid binding protein 4 (Fabp4)-Cre transgenic mice, which express Cre recombinase under the control of the Fabp4 promoter (Fig. S5). Consistent with the results obtained from human SGBS adipocytes, IFNβ treatment induced aromatase expression and E_2_ production in the primary culture of preadipocytes isolated from the inguinal mammary gland of the Ifi204^f/f^ mice. However, the increases in aromatase expression and E_2_ production were far lower in the preadipocytes from the Ifi204-AKO mice (Fig. 6a). Similar to the case of SGBS cells, aromatase expression and E_2_ production were significantly increased in the Ifi204^f/f^ preadipocytes when cultured with CM obtained from cocultured plates of isolated mouse bone marrow-derived macrophages (BMDM) and E0771 cells, which are mouse ER-positive mammary tumor cells [31]. However, those increases were not seen in the preadipocytes from the Ifi204-AKO cultured in the same condition (Fig. 6b). Furthermore, growth of E0771 cells increased when cocultured with Ifi204^f/f^ preadipocytes and BMDM, but it was far less with Ifi204-AKO preadipocytes. Simultaneously, expression levels of cyclin D1, pS2, and c-Myc were enhanced when cocultured with Ifi204^f/f^ preadipocytes/BMDM, but not with Ifi204-AKO preadipocytes/BMDM (Fig. 6c).

### IFI16 in adipocytes enhances obesity-associated progression of ER-positive BC in vivo

**Fig. 7:**
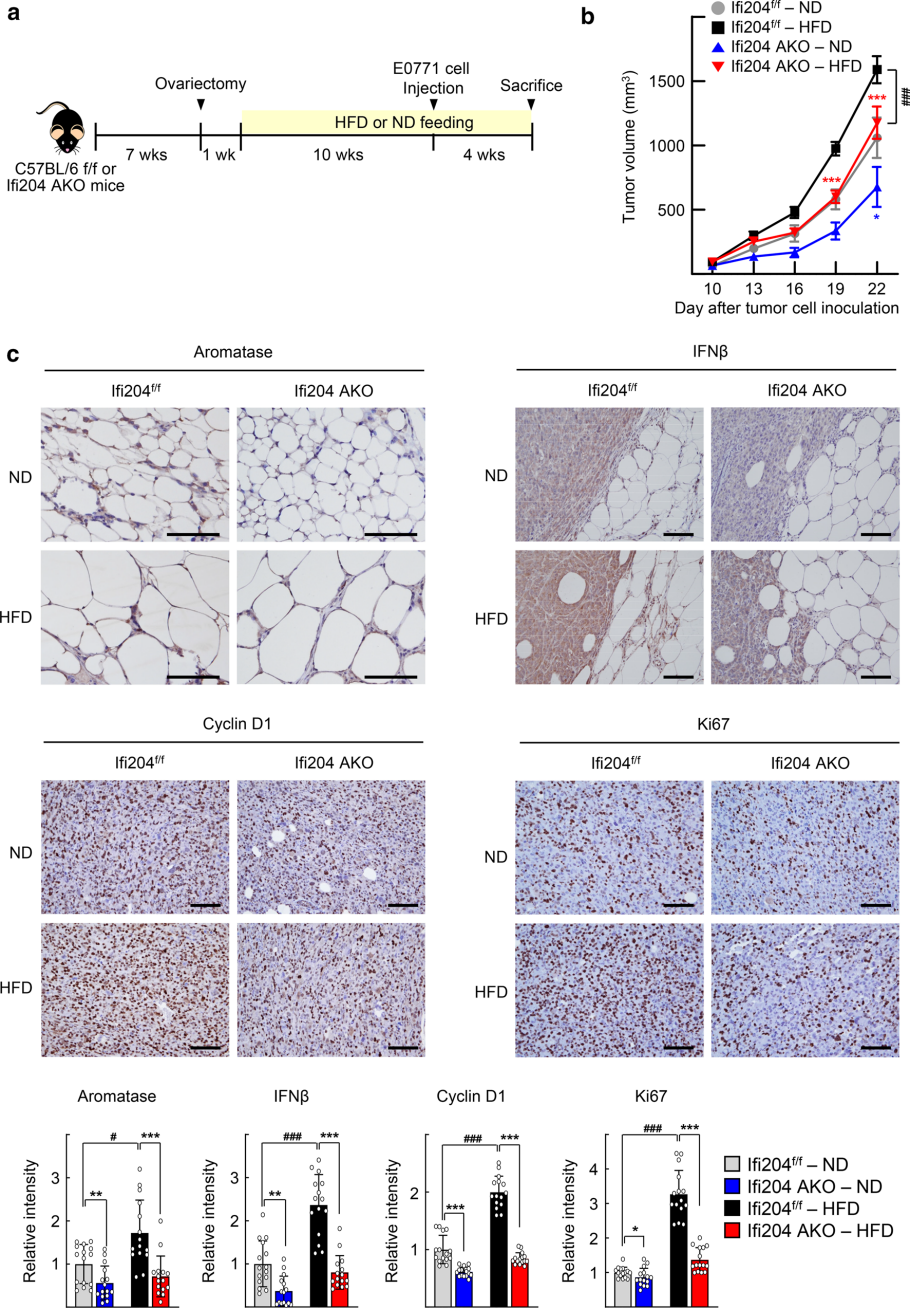
Adipocyte-specific knockdown of Ifi204 suppresses growth of E0777 mouse mammary tumor cells in allograft experiments. **a** Schematic representation of the mouse allograft experiment. Ifi204-AKO and Ifi204^f/f^ mice were ovariectomized at the age of 7 weeks. One week after ovariectomy, mice were fed with either high-fat diet (HFD) or normal diet (ND) for 10 weeks, and then E0777 cells were implanted into the mammary fat pad of mice. Mice were continuously fed with either HFD or ND until the end of the experiment. **b** Tumor volume was measured every 3 days with a caliper. The number of specimen of each group was as follows: Ifi204^f/f^-ND (*n* = 8), Ifi204^f/f^-HFD (*n* = 12), Ifi204-AKO-ND (*n* = 6), and Ifi204-AKO-HFD (*n* = 12). Data are presented as the mean ± SEM. Statistical analysis was performed using two-way ANOVA followed by the Bonferroni posttest. **P < 0.01 and ***P < 0.001 vs Ifi204^f/f^ mice on the same diet at each time point, ^###^*P* < 0.001 vs Ifi204^f/f^ mice with HFD. **c** Immunohistochemistry staining of aromatase, IFNβ, Cyclin D1, and Ki67 in tumor sections. Representative images from each group are shown (top). Staining intensities were quantified from three tumor samples from each group and five random fields per sample using ImageJ software and the IHC Profiler plug-in (bottom). **P < 0.01 and ***P < 0.001 vs Ifi204^f/f^ mice on the same diet, ^#^*P* < 0.05 and ^###^*P* < 0.001 as indicated. Bar represents 100 μm

To examine the effect of Ifi204 depletion on the ER-positive breast tumor growth in vivo, E0771 tumor cells were orthotopically inoculated in the ovariectomized Ifi204-AKO mice to avoid systemic E_2_ production (Fig. 7a). The E0771 tumor growth was reduced in the Ifi204-AKO mice compared with that in the Ifi204^f/f^ mice. The growth difference became significant when the mice were fed a high-fat diet (HFD) (Figs. 7b and S6). Immunohistochemical staining of the mammary tumor tissues showed the expression of ERα proteins in the tumor tissues (Fig. S7). Aromatase expression was significantly increased in cancer-associated adipocytes of the HFD-fed mice compared with that in cancer-associated adipocytes of normal diet-fed mice. However, such increase was not observed in the Ifi204-AKO mice. In addition, IFNβ was highly expressed both in tumor cells and infiltrated immune cells of the HFD-fed control mice. However, the level of IFNβ was significantly lower in the AKO mice. The levels of cyclin D1, one of the downstream targets of ER signaling, and Ki67, a proliferation marker, greatly increased after HFD feeding in the control mice, but the levels were significantly low in the Ifi204-AKO mice (Fig. 7c).

**Fig. 8:**
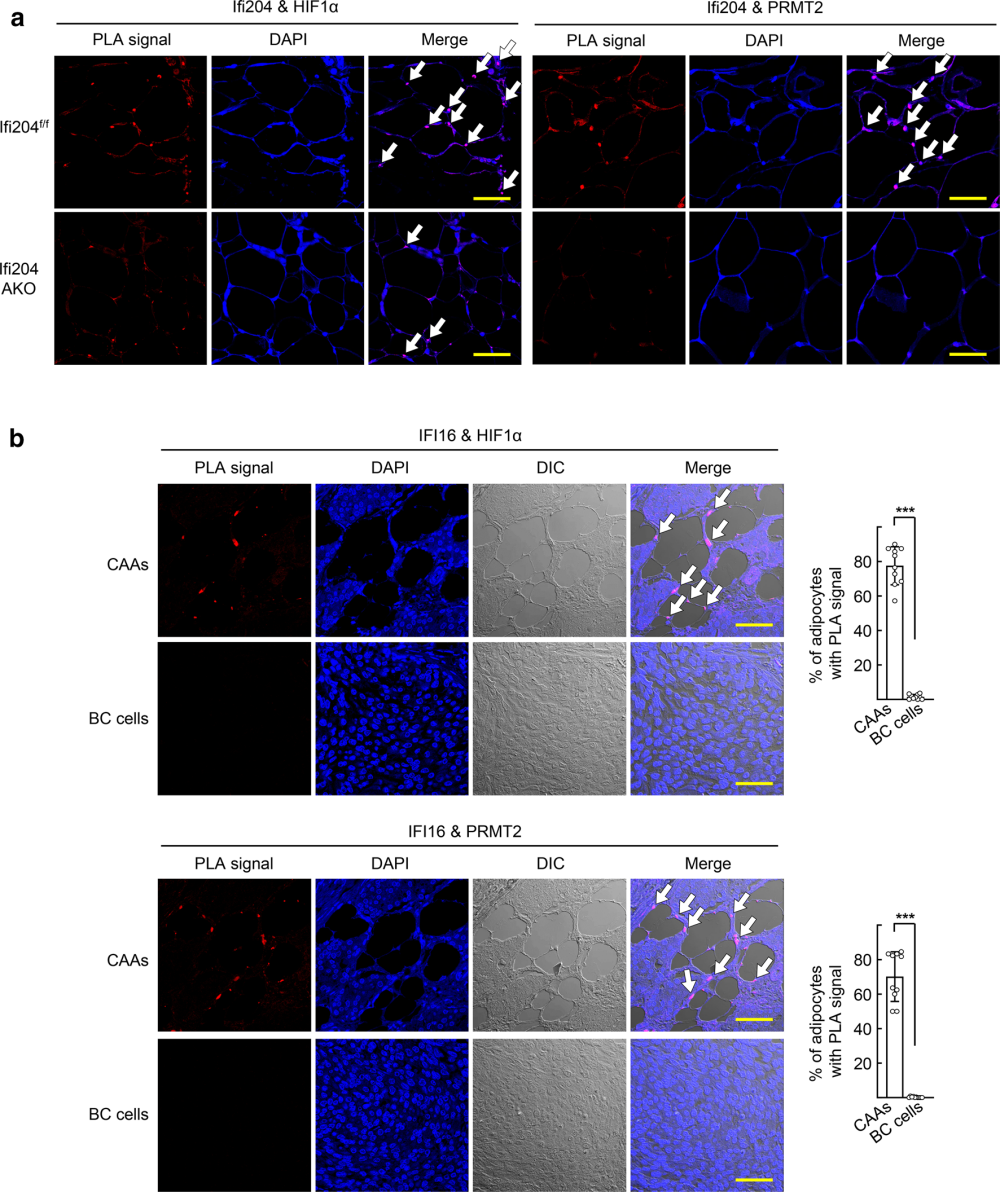

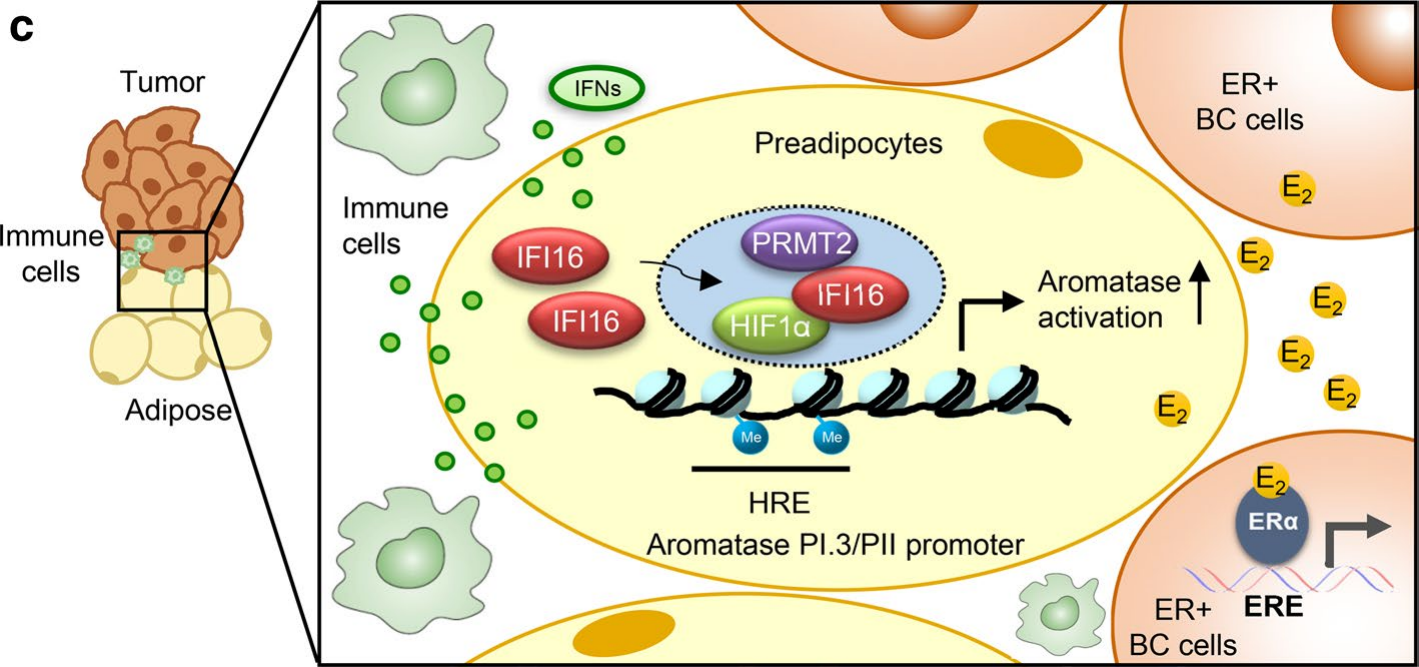
IFI16, HIF1α, and PRMT2 are physically associated in cancer-associated adipocytes of mouse allograft tumors and human BC specimens. **a** Allograft tumor tissue sections from HFD-fed mice were subjected to proximity ligation assay (PLA) using anti-Ifi204 and HIF1α antibodies (left), and anti-Ifi204 and PRMT2 antibodies (right), respectively. Arrows indicate PLA signals detected in the nucleus of adipocytes adjacent to mammary tumor cells. Quantification of the PLA signals are shown in Fig. S8. Bar represents 50 μm. **b** Luminal-type BC tissue sections were subjected to PLA using anti-IFI16 and HIF1α antibodies (top), and anti-IFI16 and PRMT2 antibodies (bottom), respectively. Cancer-associated adipocytes (CAAs) and BC cells were analyzed from the same specimens. Arrows indicate PLA signals detected in the nucleus of CAA. The number of cells with PLA-positive signal was counted from ten BC specimens (right). Data are presented as the mean ± SD. ***P < 0.001. Bar represents 50 μm. **c** Schematic model for the role of type I IFNs in aromatase activation and E_2_-dependent growth of ER-positive BC cells. The level of type I IFNs are elevated through the interaction between immune cells and BC cells, which induces the expression of IFI16 in preadipocytes. IFI16 then binds to the HRE site on the aromatase PI.3/PII promoter together with HIF1α and PRMT2, leading to the activation of aromatase transcription. Abundant estradiol in tumor microenvironment promotes growth of ER-positive BC cells

Finally, we confirmed the physical interactions of Ifi204, HIF1α, and PRMT2 in the tumor tissues using PLA. Interaction signals of Ifi204 with HIF1α and that with PRMT2 were clearly observed in almost 80% of adipocytes adjacent to mammary tumor cells. However, the interaction signals were rarely detected in tissue sections from Ifi204-AKO mice (Figs. 8a and S8). Importantly, these IFI16-HIF1α and IFI16-PRMT2 interactions were observed in human luminal-type BC specimens. The interaction signals were detected in cancer-associated adipocytes, but not in BC cells, probably due to the low expression of IFI16 in luminal-type BC cells as demonstrated previously (Fig. 8b) [13].

Taken together, these findings demonstrate that elevated levels of type I IFNs in the breast tumor microenvironment led to IFI16-mediated aromatase activation and E_2_ production in adipose tissue surrounding BC, supporting the E_2_-dependent growth of ER-positive BC cells (Fig. 8c).

## Discussion

Decades of research have demonstrated that IFNs exert a wide range of antitumor activities, including induction of apoptosis, inhibition of angiogenesis and proliferation, cell terminal differentiation, and immune regulation [25]. Moreover, recently observed immunotherapeutic benefit of IFNs in combination with PD1-targeted therapies appeared promising to overcome aggressive TNBC [12]. However, protumoral properties have also been described for IFNs [14–16]. Until now, the mechanisms underlying the differential effects of type I IFNs have not been clearly understood. In this study, we found that IFNα and IFNβ alone did not affect the growth of cancer cells, but they enhanced cancer cell growth when the cells were cocultured with preadipocytes (Figs. 5e and 6c). This observation highlights the importance of tumor microenvironmental context in the determination of anti- or protumorigenic effects of type I IFNs. Adipose tissue is the most abundant stromal component in BC, which is further increased with obesity. Thus, protumorigenic effects of type I IFNs that stimulate estrogen production in cancer-associated adipocytes may be more profound, particularly in postmenopausal patients with BC. Furthermore, tumor infiltration of CD8^+^ T cells, which is a critical factor for type I IFN-elicited antitumor immunity, was reported to be lower in ER-positive BC than in other immunogenic tumor types [32]. This low immunogenic property may also contribute to the protumorigenic effect of type I IFNs observed in ER-positive BC.

IFI16 is well recognized as an innate immune sensor that binds to foreign DNA and induces cGAS/STING-mediated IFNβ pathway signaling, but its role as a transcriptional regulator is not well characterized [33, 34]. IFI16 was shown to bind dsDNA in a non-sequence specific manner through electrostatic attraction between the HIN domain residues and the sugar-phosphate backbone of dsDNA [35]. Thus, recruitment of IFI16 to specific DNA regulatory sequences may require specific transcription factors and epigenetic modifiers. In fact, IFI16 interacted with p53 at the protein level and thereby modulated recruitment of p53 to a specific promoter to enhance the expression of its target gene, p21 [36, 37]. Here, we demonstrated that IFI16 was recruited to the HRE of the aromatase gene promoter in an HIF1α-dependent manner (Fig. 4g). In addition, we showed that IFI16 recruited PRMT2 to the HRE, which led to an increased level of H3R8me2a (Fig. 4d and g). The PRMT2-mediated H3R8me2a modification is known to play a critical role in establishing and maintaining active histone marks, such as H3K4me1, H3K4me3, and H3K27ac [38]. Thus, IFI16-induced PRMT2 recruitment and deposition of H3R8me2a may open chromatin and facilitate robust assembly of the HIF1α–IFI16–PRMT2 complex. Together with a recent observation that IFI16 was methylated by PRMT5, our findings suggest close crosstalk between IFI16 and PRMT family genes in epigenetic regulation of target gene expression [39]. Further investigations including identification of genome-wide targets of IFI16 may provide a deeper understanding of the role of IFI16 in transcriptional regulation and epigenetic modification with regard to E_2_ signaling. To assess the clinical relevance of our findings, we showed the physical interaction of HIF1α and PRMT2 in cancer-associated adipocytes of human luminal-type BC specimens obtained commercially (Fig. 8b). However, the number of samples and types of BC were very limited to expand our findings to pathogenesis of human BC. Further studies employing a large cohort of human BC are needed to confirm our findings and to provide better insight into the pathophysiological functions of type I IFNs in BC microenvironment.

Aromatase inhibitors have been proven to be highly effective and are currently the first-line endocrine therapy for postmenopausal women with ER-positive BC [40]. However, they cause severe estrogen deprivation throughout the body, resulting in a variety of side effects, such as reduction of bone density, musculoskeletal pain, abnormal lipid metabolism, and cardiovascular toxicity [41]. Therefore, blocking the transcription of the aromatase gene specifically in the adipose tissue surrounding BC could avoid such undesirable side effects of systemic suppression and could replace or supplement the existing aromatase inhibitors. Few pharmacological agents that suppress the BC-specific and adjacent adipose tissue-specific PI.3/PII-driven aromatase transcripts have been investigated in preclinical and clinical studies. For example, a histone deacetylase inhibitor, panobinostat, reduced aromatase expression and suppressed proliferation of ER-positive BC cells when combined with the aromatase inhibitor, letrozole [42]. SIRT1/2 inhibitors and sodium butyrate reduced aromatase expression in human BC cells and breast adipose fibroblasts, respectively [43, 44]. Metformin inhibited aromatase expression and thus the potential efficacy of a combination of metformin and letrozole was evaluated in a phase II randomized clinical trial in postmenopausal patients with ER-positive BC (NCT01589367) [45, 46]. Here, we showed that blocking of IFNα and/or IFNβ using anti-IFNα and/or anti-IFNβ antibodies significantly attenuated the level of aromatase in SGBS cells (Fig. S2d). This finding may aid development of a novel therapeutic strategy against ER-positive BC. This strategy could have clinical potential, because two phase III clinical trials of anifrolumab, which is a monoclonal antibody against the type I IFN receptor, for the treatment of systemic lupus erythematosus have recently been completed (NCT02446912 and NCT02446899) [47]. Together, the strategies that inhibit type I IFN signaling in BC microenvironment may be established especially for postmenopausal patients in near future.

## Materials and methods

### Cohort analysis of BC patients based on public datasets

The GSE21653 and the GSE6532 datasets were obtained and analyzed in the CTGS website (http://ctgs.biohackers.net/) [48]. The GSE21653 dataset provided the status of ER, progesterone receptor (PR), and erb-b2 receptor tyrosine kinase 2 (ERBB2), those were determined by immunohistochemistry (IHC). ER + group was defined by the positive for ER. TNBC group was defined by negative for ER, PR, and ERBB2. In the GSE6532 dataset, ER status was determined by ESR1 expression. The GSE22219 dataset was downloaded from NCBI Gene Expression Omnibus (GEO; http://www.ncbi.nlm.nih.gov/geo/) and the E-TABM-158 dataset was downloaded from ArrayExpress (http://www.ebi.ac.uk/arrayexpress/). Data that lacked expression signals in the microarrays or without clinical information records were excluded from all analyses. The processed data including normalization procedures were obtained from the corresponding websites, and no additional transformations were performed.

### Cell culture and reagents

MCF7, T47D, BT474, and THP1 cells were obtained from American Type Culture Collection. E0771 cells were obtained from CH3 Biosystems. The human preadipocyte cell strain, Simpson–Golabi–Behmel syndrome (SGBS), was kindly provided by Dr. Martin Wabitsch (University of Ulm) [23]. MCF7 and BT474 cells were maintained in Dulbecco’s modified Eagle’s medium (DMEM) supplemented with 10% fetal bovine serum (FBS; SH30084.03, Hyclone). T47D, THP1, and E0771 cells were maintained in RPMI-1640 medium supplemented with 10% FBS (SH30084.03, Hyclone). SGBS cells were maintained in DMEM‐F12 (1:1) medium containing 10% FBS (#26140-079, Gibco), 33 μM biotin (Merck), and 17 μM pantothenate (Merck). The cells were grown in an incubator with 5% CO_2_/95% air at 37 °C and routinely certified free of mycoplasma contamination using the MycoAlert Mycoplasma Detection Kit (Lonza).

Coculture of MCF7 and THP1 was performed using a transwell coculture system. THP1 cells were seeded in the bottom chambers of the transwell and then treated with 100 ng/ml phorbol 12-myristate 13-acetate (PMA) for 48 h. Then, MCF7 cells were seeded on the polyester membrane inserts with 0.4 μM pore size with free exchange of medium. After additional 96 h, conditioned medium was harvested, centrifuged and supernatants were frozen at -80 ℃ for further analyses. For THP1, SGBS, and MCF7 triple coculture experiments, THP1 cells, pre-treated with 100 ng/ml PMA for 48 h, and SGBS cells were seeded together in the bottom chambers of the transwell. Then, MCF7 cells were seeded in the top chamber of the transwell insert and cultured for 1 to 5 days. The medium was changed every 2 days.

Recombinant human IFNα and IFNβ, and murine IFNβ were purchased from R&D systems. Neutralizing antibodies against IFNα (#31110-1) and IFNβ (#31401-1) were purchased from PBL Assay Science.

### Plasmids, siRNA duplexes, and transient transfection

Myc-tagged IFI16 or HIF1α was constructed by inserting a PCR-amplified full-length human IFI16 or HIF1α cDNA into pCMV-Myc. HA-PRMT2 was kindly provided by Dr. Seung-Hoi Koo (Korea University, Seoul, South Korea). siRNA duplexes targeting IFI16, IFIT1, IFI27, IFIX, MNDA, AIM2, PRMT2, HIF1α, Ifi204, and the gene that encodes nonspecific green fluorescent protein (GFP) were synthesized and purified by Bioneer Co. Ltd (Table S1). Transient transfection of plasmids and siRNA was performed using TransIT-X2 transfection reagent (Mirus Bio) and Lipofectamine 2000 (Thermo Fisher Scientific), respectively, as described previously [49].

### Western blotting, coimmunoprecipitation, in situ proximity ligation assay, and ELISA

Western blotting and coimmunoprecipitation were performed as described previously using specific antibodies against aromatase (#677; kindly provided by Dr. Dean P. Edwards (Baylor College of Medicine), and sc-14245, Santa Cruz Biotechnology), IFI16 (sc-8023, Santa Cruz Biotechnology), Actin (sc-1616), HIF1α (sc-10790), PRMT2 (ab154154, Abcam), and Ifi204 (NBP2-27153, Novus Biologicals) [49]. To detect protein–protein interactions with high selectivity and sensitivity, PLAs were performed using the Duolink In Situ Red Starter Kit Mouse/Rabbit (Sigma Aldrich) according to manufacturer’s protocol. Briefly, cells were fixed and incubated with primary antibodies against IFI16 (sc-8023, Santa Cruz Biotechnology), HIF1α (sc-10790), and PRMT2 (ab154154, Abcam). The cells were incubated with PLA probes and the subsequent ligation and rolling circle amplification were performed. The PLA signals were visualized using a Zeiss LSM 710 confocal microscope (Carl Zeiss).

The amounts of IFNα, IFNβ, and IFNγ protein were measured using commercial ELISA kits (MBS2506739, MBS2513798, and MBS2512904, respectively, MyBioSource) according to the manufacturer’s protocol. The amount of estradiol was measured using Estradiol ELISA kit (#582251 and #501890, Cayman) according to the manufacturer’s protocol.

### Quantitative real-time polymerase chain reaction (qPCR)

Total RNA was extracted from cells using easy-BLUE™ Total RNA Extraction Kit (iNtRON Biotechnology) according to the manufacturer’s protocol. cDNA was synthesized from 2 μg of the total RNA using M-MLV reverse transcriptase (Thermo Fisher Scientific) with random hexamer (Thermo Fisher Scientific). qPCR was performed using SYBR Green PCR Master Mix (Thermo Fisher Scientific) and specific primers (Table S1) in the StepOnePlus Real-Time PCR System (Thermo Fisher Scientific). The relative mRNA level of target gene was analyzed by the equation 2^− Δ*Ct*^ (Δ*Ct* = *Ct* of target gene minus *Ct* of β-actin). Data were presented as fold induction relative to control group [50].

### Chromatin immunoprecipitation (ChIP) assay

Cells were crosslinked with 0.75% formaldehyde for 15 min at room temperature and then quenched by adding glycine to the final concentration of 0.125 M. The cells were harvested, lysed in cell lysis buffer (50 mM Tris–HCl pH 7.5, 150 mM NaCl, 5 mM EDTA, 0.5% NP40, 1% Triton X-100, and protease inhibitor), and centrifuged at 13,000 rpm for 1 min at 4 °C. The pellets containing nuclei were resuspended in lysis buffer (50 mM HEPES–KOH pH 7.5, 140 mM NaCl, 1 mM EDTA, 1% Triton X-100, 0.1% sodium deoxycholate, 0.1% SDS, and protease inhibitor). Nuclear lysates were sonicated using the Branson 102C sonicator and centrifuged. The supernatants containing chromatin were collected and 10% the supernatant was saved as input. For immunoprecipitation, the rest of supernatants were incubated with antibodies against IFI16 (sc-8023, Santa Cruz Biotechnology), Myc (sc-40), HA (sc-805), H3R8me2a (NB21-1062, Novus Biologicals) or a control IgG antibody (Santa Cruz Biotechnology) overnight at 4 °C. The following day, protein G or protein A agarose (Millipore) that had been blocked with the herring sperm DNA and BSA was added and incubated for 2 h at 4 °C. The immunoprecipitates were washed five times, eluted with elution buffer (1% SDS and 100 mM NaHCO_3_), and digested with proteinase K (GenDEPOT) at 65℃ overnight. DNA fragments were purified using phenol∶chloroform∶isoamyl alcohol extraction and ethanol precipitation. The purified DNA fragments were subjected to amplification by PCR or qPCR using specific primers (Table S1). Data were normalized to input and presented as fold enrichment relative to the IgG control.

### Generation of CRISPR/Cas9-mediated IFI16 knockout cell lines

To generate IFI16 KO cell lines, SGBS cells were transfected with 1 μg of IFI16 CRISPR/Cas9 KO plasmid (sc-416568, Santa Cruz Biotechnology) or a control CRISPR/Cas9 plasmid (sc-418922) using Lipofectamine 2000 (Thermo Fisher Scientific) according to manufacturer’s protocol. Two days after transfection, GFP-positive cells were sorted by FACS Aria (BD Biosciences) and seeded as single cells in 96-well plates. Clones were expanded and subsequently confirmed IFI16 KO by western blotting.

### Generation of adipocyte-specific Ifi204^−/−^ mice

Animal experiments were approved by Seoul National University Institutional Animal Care and Use Committee. The Ifi204^tm1a(KOMP)Wtsi^ heterozygous mice were obtained from the Knockout Mouse Project (KOMP) repository at the University of California, Davis. The Ifi204^tm1a(KOMP)Wtsi^ mice were bred with the FLP deleter strain B6N(B6J)-Tg(CAG-Flpo)1Afst/Mmucd (036512-UCD, MMRRC) to excise the lacZ-neo cassette to generate the conditional allele Ifi204^tm1c(KOMP)Wtsi^ (referred to in the text as Ifi204^f/f^). To produce the adipocyte-specific Ifi204 KO line (Fabp4^cre^-Ifi204^f/f^), Ifi204^f/f^ mice were bred with B6N.Cg-Tg(Fabp4-cre)1Rev/J mice (#018965, The Jackson Laboratory), which express Cre recombinase under the control of the mouse fatty acid binding protein 4 (Fabp4) promoter (Fig. S5a). Offspring were genotyped to confirm the inclusion of loxP sites within Ifi204 allele and the presence of Cre recombinase via PCR using specific primers (Fig. S5b and Table S1). All animals were maintained in an air-conditioned room at a temperature of 22–24 °C and humidity of 37–64%, with a 12-h light/dark cycle.

### Isolation and culture of primary preadipocytes and BMDMs

For primary preadipocyte culture, stromal vascular fractions from the inguinal mammary gland were obtained from female mice. Briefly, mammary gland tissues were minced and digested with 2 mg/ml collagenase type I (Sigma Aldrich) in Krebs–Ringers bicarbonate buffer (Sigma Aldrich) containing 1% BSA for 1 h at 37 °C. Digested samples were passed through a sterile 100 μm cell strainer and centrifuged at 500*g* for 5 min at 4 °C. The pellets containing the stromal vascular fraction were resuspended in red blood cell lysing buffer (Sigma Aldrich) and then collected by centrifugation at 500*g* for 5 min at 4℃. The pellets were washed twice in complete culture medium and subjected to cell culture.

BMDMs were isolated from the femur and tibia of wild-type mice. The marrow was passed through a 40 μm strainer and centrifuged at 1800 rpm for 7 min at 4℃. The pellets were resuspended in red blood cell lysing buffer, incubated for 10 min at room temperature, and then collected by centrifugation. Cells were plated in DMEM supplemented with 10% FBS, 1% penicillin/streptomycin, 1 × MEM non-essential amino acids solution (Gibco), 1 mM Sodium pyruvate, 0.25 mM β-mercaptoethanol, and 20 ng/ml macrophage colony-stimulating factor, and allowed differentiation for 5 days.

For coculture of BMDM and E0771 cells, BMDMs were seeded in the bottom chambers of the transwell and E0771 cells were seeded on the polyester membrane inserts with 0.4 μM pore size with free exchange of medium. After additional 48 h, conditioned medium was harvested, centrifuged and supernatants were frozen at − 80 °C for further analyses. For BMDM, primary preadipocytes, and E0771 triple coculture experiments, BMDMs and primary preadipocytes were seeded together in the bottom chambers of the transwell. Then, E0771 cells were seeded in the top chamber of the transwell insert and cultured for 1–5 days. The medium was changed every 2 days.

### Allograft experiments and histological analyses

For the orthotopic transplantation tumor models, female Ifi204-AKO and Ifi204^f/f^ mice were ovariectomized at the age of 7 weeks. One week after ovariectomy, mice were fed with either HFD (D12492, Research Diets) or ND (D12450J) for 10 weeks. E0771 (1 × 10^5^) cells mixed 1:1 with Matrigel (BD Biosciences) were injected into the mammary fat pad of mice. Mice were continuously fed with either HFD or ND until the end of the experiment. Body weight was monitored weekly. Tumor diameter was measured every 3 days with a caliper and the tumor volume was calculated using the following formula: tumor volume (mm^3^) = width^2^ × length × 0.5.

For immunohistochemistry (IHC) or proximity ligation assay (PLA) of the mouse mammary tumor tissues, slides of tumor sections were deparaffinized and processed for antigen retrieval. IHC was performed using specific antibodies against aromatase (ab18995, Abcam), IFNβ (PA5-20390, Thermo Fisher Scientific), Cyclin D1 (ab16663), Ki67 (ab16667), and ERα (sc-7207, Santa Cruz Biotechnology), as described previously [49]. The sections incubated without primary antibody were used as the negative control. Staining intensity was quantified by densitometric analysis using ImageJ software and the IHC Profiler plug-in (https://sourceforge.net/projects/ihcprofiler/). PLA was performed using antibodies against Ifi204 (NBP2-27153, Novus Biologicals), HIF1α (NB100-131), and PRMT2 (sc-390089, Santa Cruz Biotechnology), and the Duolink In Situ Red Starter Kit Mouse/Rabbit (Sigma Aldrich) according to manufacturer’s protocol.

### Human breast cancer tissue microarray

Luminal A type breast cancer tissue array slides (BR1507) were purchased from US Biomax. For PLA of breast cancer tissues, slides were deparaffinized, processed for antigen retrieval, and then subjected to PLA using antibodies against IFI16 (sc-8023, Santa Cruz Biotechnology), HIF1α (sc-10790), and PRMT2 (ab154154, Abcam).

### Statistical analyses

Statistical analyses were performed using GraphPad Prism software. Experimental values were expressed as the mean ± standard deviation (SD) based on three independent experiments, unless indicated otherwise. Statistically significant differences between two groups were determined using the nonparametric Mann–Whitney *U* test. Statistical analyses of multiple groups were performed using two-way ANOVA followed by the Bonferroni posttest. *P* < 0.05 was considered significantly different.

### Supplementary Information

Below is the link to the electronic supplementary material.

Supplementary file1 (PDF 1185 KB)

## Data Availability

The GSE21653 and the GSE6532 datasets were obtained and analyzed in the CTGS website (http://ctgs.biohackers.net/) [48]. The GSE22219 dataset analyzed during the current study is available in the NCBI Gene Expression Omnibus (GEO; http://www.ncbi.nlm.nih.gov/geo/). The E-TABM-158 dataset analyzed during the current study is available in the ArrayExpress (http://www.ebi.ac.uk/arrayexpress/).
