# Supplementary material for: Type I IFN stimulates IFI16-mediated aromatase expression in adipocytes that promotes E2-dependent growth of ER-positive breast cancer
Source: Cell Mol Life Sci. 2022 May 20;79(6):306. doi: 10.1007/s00018-022-04333-y (PMC9122892; doi:10.1007/s00018-022-04333-y)
Supplement: Supplementary file 1 — Supplementary file1 (PDF 1185 KB) [file 18_2022_4333_MOESM1_ESM.pdf]

Supplementary Information

Type I IFN stimulates IFI16-mediated aromatase expression in adipocytes that promotes E_2-dependent growth of ER-positive breast cancer

Na-Lee Ka[1,2], Ga Young Lim[1], Seung-Su Kim[1], Sewon Hwang[1], Juhyeong Han[1], Yun-Hee Lee[1,2], and Mi-Ock Lee[1,2,3,*]

[1]College of Pharmacy, Seoul National University, Seoul 08826, South Korea.

[2]Research Institute of Pharmaceutical Sciences, Seoul National University, Seoul 08826, South Korea.

[3]Bio-MAX institute, Seoul National University, Seoul 08826, South Korea.

[*]Corresponding author. Tel: +82 2 880 9331; E-mail: molee@snu.ac.kr

Supplementary Table S1. Oligonucleotide sequences used in the present investigation.

Gene	Accession number		Nucleotide sequence	Species	Purpose
IFI16	NM_001206567.2	sense	5'-GCUGACCGAAACAUGGAGA-3'	Human	siRNA
IFIT1	NM_001548.5	sense	5'-UACAUGGGAGUUAUCCAUUGA-3'	Human	
IFI27	NM_001130080.3	sense	5'-CUGAAGUUGAGGAUCUCUU-3'	Human	
IFIX	NM_152501.5	sense	5'-GGAGUAAGAUGUCCAAAGA-3'	Human	
MNDA	NM_002432.3	sense	5'-CUCUGAAUGUAAAGGAGUA-3'	Human	
AIM2	NM_004833.3	sense	5'-GUCAGAGUCUUUGUAGCUU-3'	Human	
PRMT2	NM_206962.4	sense	5'-CUGACAUCAUCACCGUGUA-3'	Human	
HIF1α	NM_001530.4	sense	5'-GUGGUUGGAUCUAACACUA-3'	Human	
GFP (Control)		sense	5'-GUUCAGCGUGUCCGGCGAG-3'		
Aromatase	NM_000103.4	sense<br>antisense	5'-ACCCTTCTGCGTCGTGTC-3'<br>5'-TCTGTGGAAATCCTGCGTCTT-3'	Human	qPCR
	NM_007810.4	sense<br>antisense	5'-ATGTTCTTGGAAATGCTGAACCC-3'<br>5'-AGGACCTGGTATTGAAGACGAG-3'	Mouse	
Aromatase PI.4		sense<br>antisense	5'-GTGACCAACTGGAGCCTG-3'<br>5'-CATGGCTTCAGGCACGAT-3'	Human	
Aromatase PI.3		sense<br>antisense	5'-CTTGCCTAAATGTCTGATCACATTA-3'<br>5'-CATGGCTTCAGGCACGAT-3'	Human	
Aromatase PII		sense<br>antisense	5'-CCCTTTGATTTCCACAGGAC-3'<br>5'-CATGGCTTCAGGCACGAT-3'	Human	
IFNα	NM_024013.3	sense<br>antisense	5'-GTGAGGAAATACTTCCAAAGAATCAC-3'<br>5'-TCTCATGATTTCTGCTCTGACAA-3'	Human	
IFNβ	NM_002176.4	sense<br>antisense	5'-GCTTCTCCACTACAGCTCTTTC-3'<br>5'-CAGTATTCAAGCCTCCCATTCA-3'	Human	
IFI16	NM_001206567.2	sense<br>antisense	5'-ACAAACCCGAGAAACAATGACC-3'<br>5'-GCATCTGAGGAGTCCGAAGA-3'	Human	
IFIT1	NM_001548.5	sense<br>antisense	5'-GATCTCAGAGGAGCCTGGCTAA-3'<br>5'-TGATCATCACCATTTGTACTCATGG-3'	Human	
IFI27	NM_001130080.3	sense<br>antisense	5'-TGCTCTCACCTCATCAGCAGT-3'<br>5'-CACAACTCCTCCAATCACAACT-3'	Human	
IFIX	NM_152501.5	sense<br>antisense	5'-GCAACCGTCTCACAGCTAAA-3'<br>5'-GAGTCTGCTCTTTGGACATCTT-3'	Human	
MNDA	NM_002432.3	sense<br>antisense	5'-GTGTCCCAAGAGCAGAGTAAG-3'<br>5'-TTGGATGGAGTTGATGATGAGG-3'	Human	
AIM2	NM_004833.3	sense<br>antisense	5'-GCCACTAAGTCAAGCTGAAATG-3'<br>5'-CAGGCTTAACATGAGGAGAGAC-3'	Human	
PRMT2	NM_206962.4	sense<br>antisense	5'-GAGTACAGCCAGAGGAGTTTG-3'<br>5'-GCAGTGGTTTGTCTCAGGATA-3'	Human	
HIF1α	NM_001530.4	sense<br>antisense	5'-TTTTTCAAGCAGTAGGAATTGGA-3'<br>5'-GTGATGTAGTAGCTGCATGATCG-3'	Human	
Cyclin D1	NM_053056.3	sense<br>antisense	5'-GGATGCTGGAGGTCTGCGA-3'<br>5'-AGAGGCCACGAACATGCAAG-3'	Human	
	NM_001379248.1	sense<br>antisense	5'-CCCTGACACCAATCTCCTCAAC-3'<br>5'-GCATGGATGGCACAATCTCCT-3'	Mouse	

pS2	NM_003225.3	sense<br>antisense	5’-ACCATGGAGAACAAGGTGAT-3'<br>5’-AAATTCACACTCCTCTTCTG-3'	Human	
	NM_009362.2	sense<br>antisense	5’-CAGGCCCAGGAAGAAACAT-3'<br>5’-CCCGGACACTGTCATCAAA-3'	Mouse	
PR	NM_001202474.3	sense<br>antisense	5'-ACAGGACCCCTCCGACGAAAA-3'<br>5'-AGCTGTCTCCAACCTTGCACC-3'	Human	
c-Myc	NM_002467.6	sense<br>antisense	5'-AAAGGCCCCCAAGGTAGTTA-3'<br>5'-GCACAAGAGTTCCGTAGCTG-3'	Human	
	NM_010849.4	sense<br>antisense	5'-TAACTCGAGGAGGAGCTGGA-3'<br>5'-GCCAAGGTTGTGAGGTTAGG-3'	Mouse	
ERα	NM_007956.5	sense<br>antisense	5'-AGAATGGCCGAGAGAGACTG-3'<br>5'-AGGCATAGTCATTGCACACG-3'	Mouse	
Ifi204	NM_008329.2	sense<br>antisense	5'-TGGAGAACACAGTTTCATCAAGATATC-3'<br>5'-ACTTGTTTGGGACCATGATGGT-3'	Mouse	
18srRNA	NR_145820.1	sense<br>antisense	5'-TGCATGGCCGTTCTTAGTTG-3'<br>5'-AGTTAGCATGCCAGAGTCTCGTT-3'	Human	
	NR_003278.3	sense<br>antisense	5'-GTAACCCGTTGAACCCCATT-3'<br>5'-CCATCCAATCGGTAGTAGCG-3'	Mouse	
PI.3/PII ChIP-1		sense<br>antisense	5'-GTTCCTGAGTGGCACCTGAG-3'<br>5'-CTCAGAGCCTTCCATCAGTT-3'	Human	ChIP
PI.3/PII ChIP-2		sense<br>antisense	5'-GGCTCTGAGAAGACCTCAACG-3'<br>5'-GCCAAATTTCTTGCCAAAT-3'	Human	
PI.3/PII ChIP-3		sense<br>antisense	5'-ACTCCACCTCTGGAATGAGCT-3'<br>5'-CTGTGGAAATCAAAGGGACAGAA-3'	Human	
PI.3/PII ChIP-3a		sense<br>antisense	5'-ACTCCACCTCTGGAATGAGCT-3'<br>5'-GTTCCTTTAGACGCTTGGTCTG-3'	Human	
PI.3/PII ChIP-3b		sense<br>antisense	5'-AGGGCAAGATGATAAGGTTCTAT-3'<br>5'-CTGTGGAAATCAAAGGGACAGAA-3'	Human	
Ifi204$^{f/f}$ allele<br>(P1 and P2)		sense<br>antisense	5'-AAGGTAACACAGAAGACCCTGTCCC-3'<br>5'-TTCTGACTCTCTGCTTGCCTCTTCC-3'	Mouse	Geno-<br>typing
aP2^{Cre} allele (Cre)		sense<br>antisense	5'-GCGGTCTGGCAGTAAAAACTATC-3'<br>5'-GTGAAACAGCATTGCTGTCACTT-3'	Mouse	

Supplementary Figures

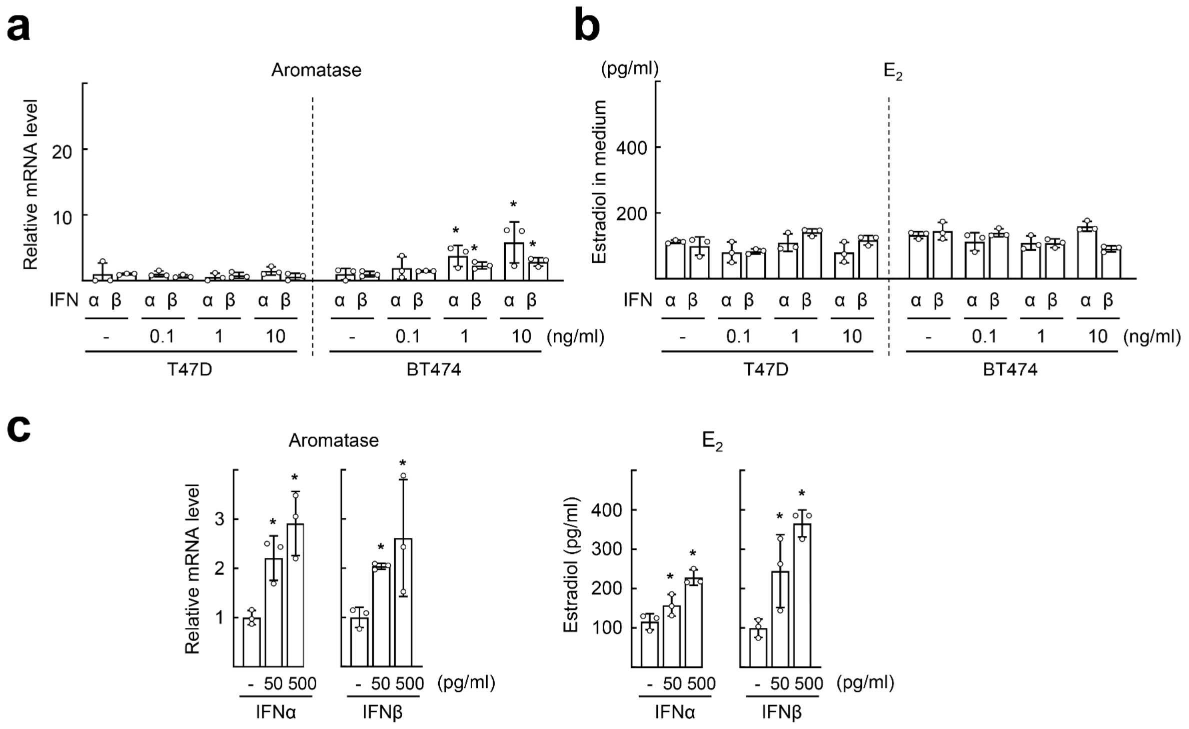

Fig. S1. Type I IFN increases aromatase expression and E_2 production in ER-positive BC cells and differentiated SGBS adipocytes. (a) T47D (4×10^5 cells) or BT474 cells (4×10^5 cells) were treated with vehicle (-), IFNα, or IFNβ for 48 h. The mRNA level of aromatase was measured by qPCR. *$P < 0.05$ *vs* vehicle (-) (n=3). **(b)** T47D (4×10^5 cells) or BT474 cells (4×10^5 cells) cells were treated with vehicle (-), IFNα, or IFNβ for 48 h, as indicated. The concentration of E_2 in the culture supernatants was measured by ELISA. **(c)** SGBS preadipocytes were induced to differentiate by incubation in serum-free DMEM/F12 medium containing 2 μM rosiglitazone, 25 nM dexamethasone, 250 μM isobutyl methylxanthine, 0.1 μM cortisol, 0.01 mg/ml transferrin, 0.2 nM triiodothyronine, and 20 nM human insulin for 4 days. The cells were then cultured in DMEM/F12 medium containing 0.1 μM cortisol, 0.01 mg/ml transferrin, 0.2 nM triiodotyronin, and 20 nM human insulin for additional 10 days. Fully differentiated cells were treated with vehicle (-), or 50 or 500 pg/ml IFNs for 48 h. The mRNA level of aromatase was measured by qPCR (left). The concentration of E_2 in the culture supernatants was measured by ELISA (right). *$P < 0.05$ *vs* vehicle (n=3).

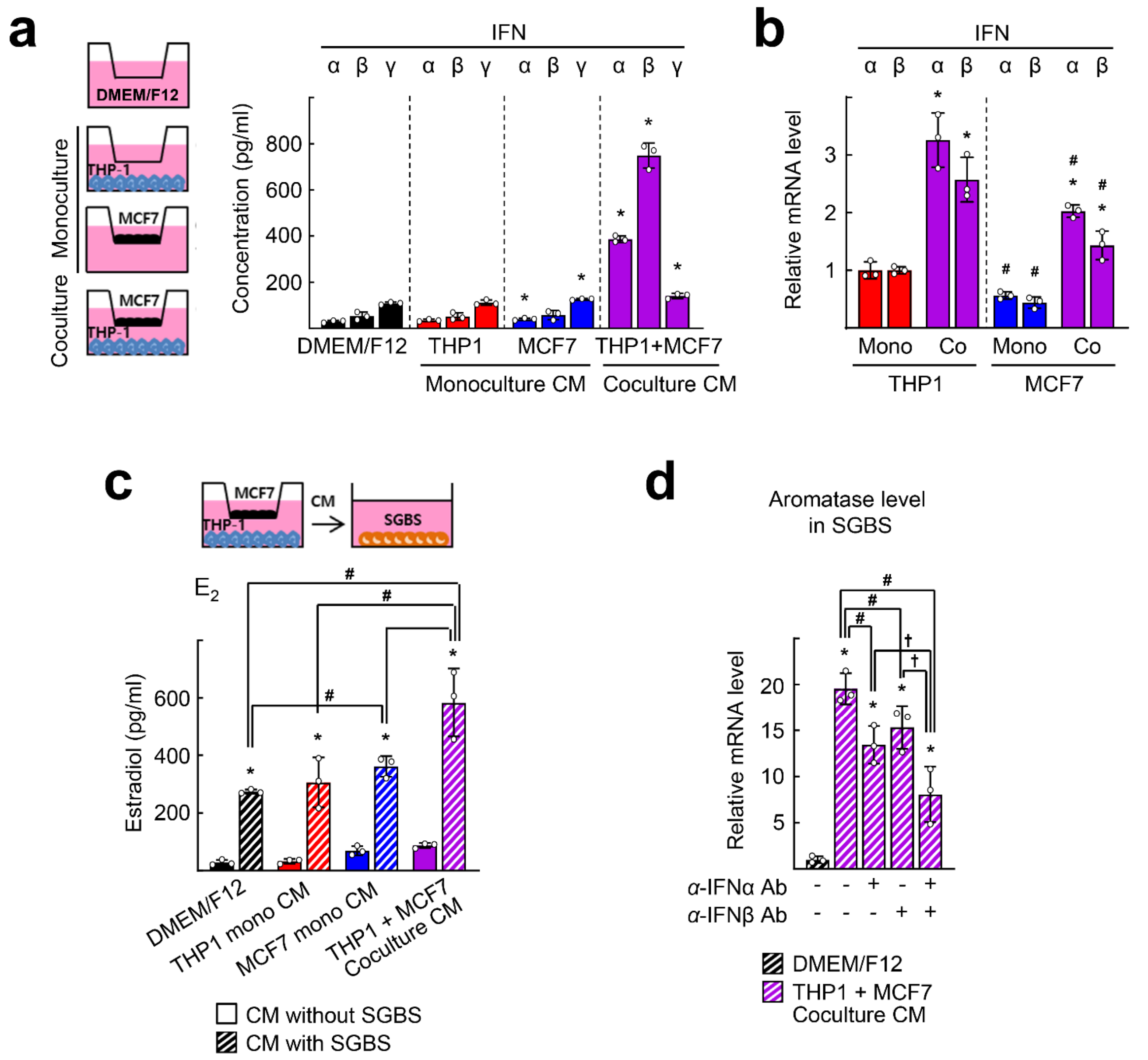

Fig. S2. Conditioned media obtained from cocultured MCF7 and THP1 increases E_2 production in SGBS preadipocytes. (a) Schematic representation of the MCF7 and THP1 coculture experiments (left). THP1 cells were seeded in the bottom chamber of a transwell and treated with 100 ng/ml PMA for 48 h. Then, MCF7 cells were seeded in the top chamber and allowed to incubate for additional 96 h. The concentration of IFNs in the culture medium was measured by ELISA. $^*P < 0.05$ *vs* DMEM/F12 medium (n=3) (right). **(b)** MCF7 and THP1 cells were monocultured (Mono) or cocultured (Co) as described in panel (a). Expression levels of IFNα and IFNβ from each type of cells were measured by qPCR. $^*P < 0.05$ *vs* monocultured cells. $^\#P < 0.05$ *vs* THP1 cells (n=3). **(c)** Schematic representation of E_2 measurement in SGBS culture media (top). MCF7 and THP1 cells were cocultured as described in panel (a). SGBS cells were incubated with conditioned medium (CM) obtained from the THP1 and MCF7 coculture for 48 h. The concentrations of estradiol in the CM incubated with or without SGBS cells were measured by ELISA $^*P < 0.05$ *vs* CM without SGBS, $^\#P < 0.05$ as indicated (n=3) (bottom). **(d)** The CM obtained from the coculture of MCF7 and THP1 cells was pre-treated with neutralizing antibodies against IFNα (5 µg/ml) and/or IFNβ (10 µg/ml) for 2 h, and supplied for the SGBS culture for 24 h. Expression level of aromatase was measured by qPCR. $^*P < 0.05$ *vs* SGBS with DMEM/F12, $^\#P < 0.05$ *vs* IgG, $^\dagger P < 0.05$ as indicated (n=3).

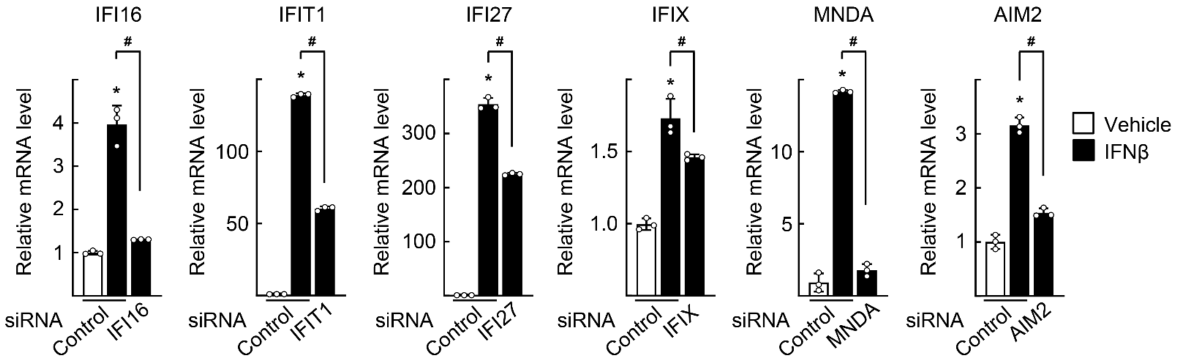

Fig. S3. Expression levels of ISGs after siRNA-mediated knockdown. Expression levels of ISGs were measured by qPCR as control for Fig. 3a. The SGBS cells were transfected with the indicated siRNAs and then treated with 10 ng/ml IFNβ for 48 h. Data are presented as the mean ± SD (n=3). **P* < 0.05 *vs* siControl with vehicle, $^{\#}P$ < 0.05 as indicated.

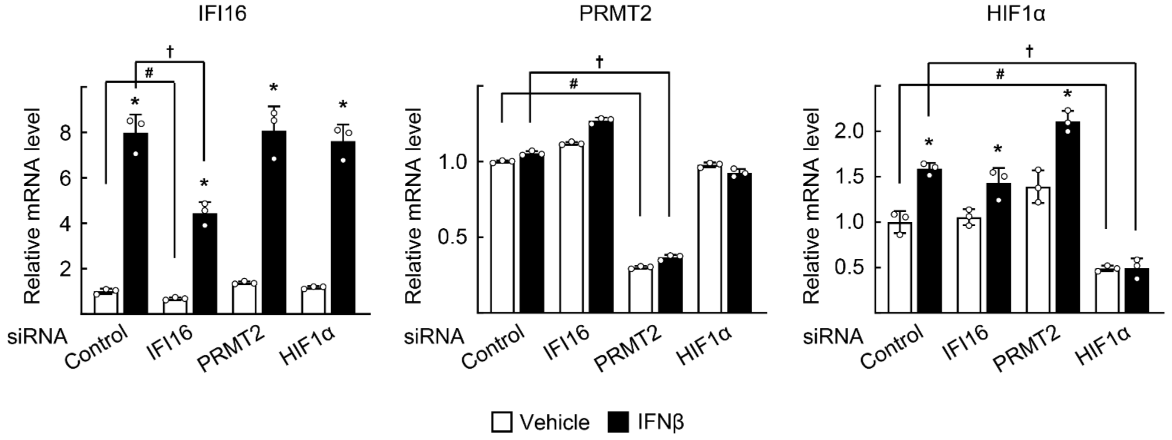

Fig. S4. Expression levels of IFI16, PRMT2, and HIF1α after siRNA-mediated knockdown. Expression levels of IFI16, PRMT2, and HIF1α were measured by qPCR as control for Fig. 4h. SGBS cells were transfected with indicated siRNAs and treated with 10 ng/ml IFNβ for 48 h. Data are presented as the mean ± SD (n=3). **P* < 0.05 *vs* vehicle, $^{\#}P$ < 0.05 *vs* si-Control with vehicle, $^{\dagger}P$ < 0.05 *vs* si-Control treated with IFNβ.

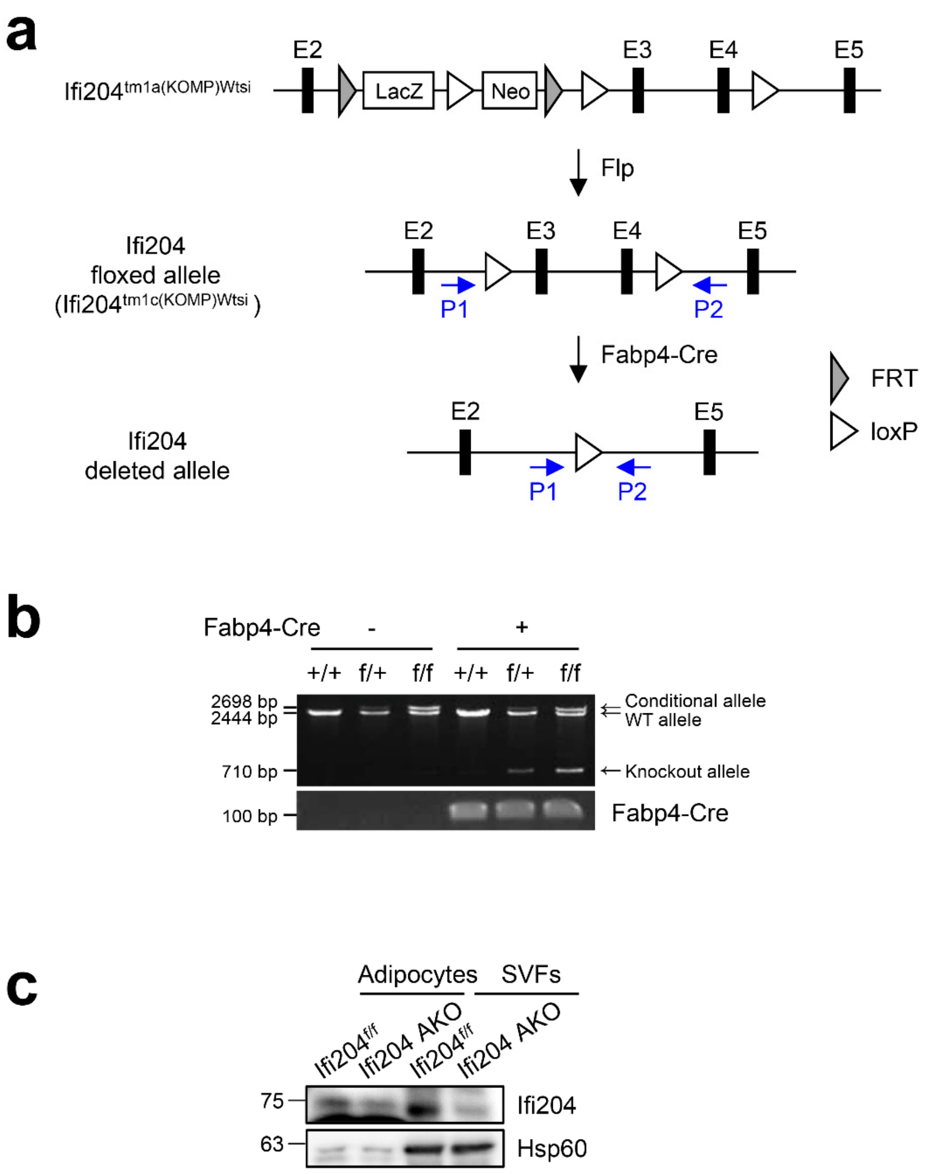

Fig. S5. Generation of adipocyte-specific Ifi204 knockout mice. (a) Schematic representation of the targeted Ifi204 floxed allele and the strategy for generation of the adipocyte-specific Ifi204-deficient allele through Fabp4-Cre recombination. E; Exon. The primer sequences (P1/P2 and Cre) used for the genotyping are shown in Table S1. **(b)** Genotyping analysis of the Ifi204$^{f/f}$ and Fabp4^{cre}-Ifi204$^{f/f}$ (Ifi204-AKO) mice. **(c)** Expression levels of Ifi204 protein in mammary gland adipocytes and stromal vascular fractions (SVFs) isolated from the Ifi204$^{f/f}$ and Fabp4^{cre}-Ifi204$^{f/f}$ mice were analyzed by western blotting.

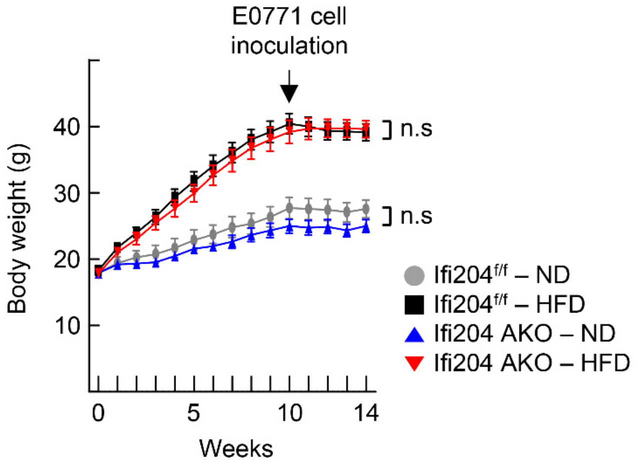

Fig. S6. Body weight of mice used in the E0771 allograft experiments. Body weight of mice used in the E0771 allograft experiments as in Fig. 7b is shown. Body weight was measured weekly after the HFD feeding. n.s; no significant difference between the groups.

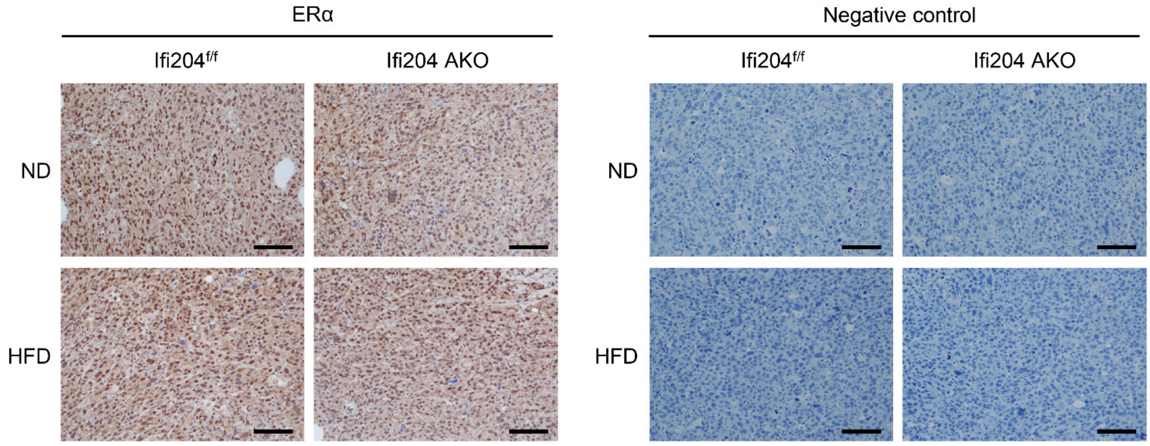

Fig. S7. Expression of ERα in the E0771 allograft tumors. E0771 allograft tumor sections were subjected to immunohistochemistry staining using ERα antibody. The sections incubated without primary antibody were used as the negative control.

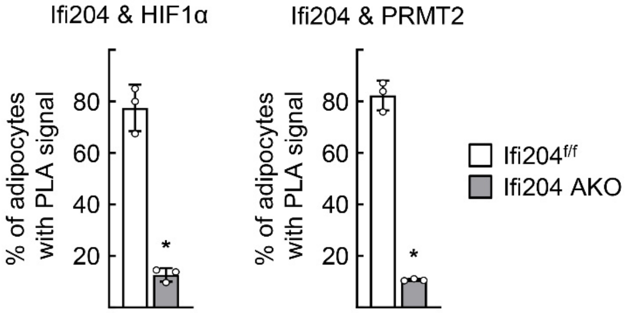

Fig. S8. Quantification of the Ifi204-HIF1α and Ifi204-PRMT2 PLA signals. The number of cells with PLA-positive signal as in Fig. 8a was counted from at least 100 cells for each sample and presented as percentage of total counted cells. Three tumor samples from each group were analyzed. Data are presented as the mean ± SEM (n=3). *P < 0.05 *vs* Ifi204$^{f/f}$ mice.
